# Studies of moss reproductive development indicate that auxin biosynthesis in apical stem cells may constitute an ancestral function for focal growth control

**DOI:** 10.1111/nph.16914

**Published:** 2020-10-04

**Authors:** Katarina Landberg, Jan Šimura, Karin Ljung, Eva Sundberg, Mattias Thelander

**Affiliations:** ^1^ Department of Plant Biology The Linnean Centre for Plant Biology in Uppsala Swedish University of Agricultural Sciences Uppsala SE‐750 07 Sweden; ^2^ Department of Forest Genetics and Plant Physiology Umeå Plant Science Centre Swedish University of Agricultural Sciences (SLU) Umeå SE‐901 83 Sweden

**Keywords:** auxin, moss, *Physcomitrium(Physcomitrella)patens*, R2D2, reproductive development, stem cell, TAR, YUC

## Abstract

- The plant hormone auxin is a key factor for regulation of plant development, and this function was probably reinforced during the evolution of early land plants. We have extended the available toolbox to allow detailed studies of how auxin biosynthesis and responses are regulated in moss reproductive organs, their stem cells and gametes to better elucidate the function of auxin in the morphogenesis of early land plants.
- We measured auxin metabolites and identified IPyA (indole‐3‐pyruvic acid) as the main biosynthesis pathway in *Physcomitrium* (*Physcomitrella*) *patens* and established knock‐out, overexpressor and reporter lines for biosynthesis genes which were analyzed alongside previously reported auxin‐sensing and transport reporters.
- Vegetative and reproductive apical stem cells synthesize auxin. Sustained stem cell activity depends on an inability to sense the auxin produced while progeny of the stem cells respond to the auxin, aiding in the control of cell division, expansion and differentiation. Gamete precursors are dependent on a certain degree of auxin sensing, while the final differentiation is a low auxin‐sensing process.
- Tha data presented indicate that low auxin activity may represent a conserved hallmark of land plant gametes, and that local auxin biosynthesis in apical stem cells may be part of an ancestral mechanism to control focal growth.

## Introduction

The plant hormone auxin is a key factor in the regulation of plant development, and this function was probably reinforced during early land plant evolution. Functional studies in the bryophyte models *Physcomitrium* (*Physcomitrella*) *patens* and *Marchantia polymorpha*, early diverging land plants separated from the flowering plants roughly 450 million years ago, lend support to this hypothesis (Rensing *et al*., 2008; Finet & Jaillais, 2012; Bowman *et al*., 2017; Thelander *et al*., 2018, 2019; Kato *et al*., 2018; Morris *et al*., 2018; Puttick *et al*., 2018). Homologs to genes encoding the nuclear auxin sensing and response machinery in the flowering plant model *Arabidopsis thaliana* are present in the model bryophyte genomes (Rensing *et al*., 2008; Flores‐Sandoval *et al*., 2015; Kato *et al*., 2015). Genetic characterization suggests strongly that the bryophyte TIR1/AFB‐AUX/IAA co‐receptors and the three classes of ARF transcription factors regulating auxin responses are functionally conserved (Ashton *et al*., 1979; Paponov *et al*., 2009; Prigge *et al*., 2010; Causier *et al*., 2012a,b; Lavy *et al*., 2016, 2012; Sugano *et al*., 2014; Eklund *et al*., 2015; Kato *et al*., 2015, 2017, 2018, 2020). In addition, PIN‐mediated directional auxin transport is important for developmental regulation, not only in flowering plants, but also in bryophytes, as demonstrated for *P. patens* (Bennett *et al*., 2014; Viaene *et al*., 2014).

Studies using exogenous auxin or bryophyte lines affected in auxin sensing, signaling or transport suggest that auxin was important for cell fate transitions early in land plant evolution. In *P. patens*, the transition from chloronema to caulonema, two filamentous tissue types, is promoted by auxin (Johri & Desai, 1973; Ashton *et al*., 1979; Cove & Ashton, 1984; Prigge *et al*., 2010; Hayashi *et al*., 2012; Lavy *et al*., 2012, 2016; Viaene *et al*., 2014; Plavskin *et al*., 2016). Likewise, the initiation of root hair‐like rhizoids is promoted by exogenous auxin and sensed through the TIR1/AFB‐AUX/IAA co‐receptors, in both bryophyte models (Ashton *et al*., 1979; Sakakibara *et al*., 2003; Prigge *et al*., 2010; Kato *et al*., 2015; Lavy *et al*., 2016). Auxin also affects branching in the bryophytes. Changes in auxin signaling by inhibition of auxin biosynthesis or overexpression (OE) of auxin responses in the *M. polymorpha* thallus caused a decreased or increased bifurcation rate, respectively (Flores‐Sandoval *et al*., 2015). In addition, reduced auxin transport in *P. patens* induces branching of the gametophore shoots and the sporophyte (Fujita *et al*., 2008; Bennett *et al*., 2014; Coudert *et al*., 2015).

During the emergence of land plants, many important characters were acquired, including three‐dimensional growth from apical meristems, which in at least flowering plants are dependent on regulated auxin sensing (Du & Scheres, 2018; Wang & Jiao, 2018). Three‐dimensional tissue growth and organ initiation occurs through divisions of a single meristematic apical stem cell in bryophytes (Harrison *et al*., 2009; Kofuji & Hasebe, 2014; Shimamura, 2016). The formation and maintenance of stem cells in *P. patens* gametophore shoots, and in *M. polymorpha* gemma are both affected by changes in auxin sensing (Bennett *et al*., 2014; Lavy *et al*., 2016; Kato *et al*., 2017). However, so far, a limited toolbox has hampered detailed studies of how auxin affects the identity and function of bryophyte apical stem cells. One additional approach is to change the levels of auxin biosynthesis. In flowering plants, the indole‐3‐pyruvate (IPyA) auxin biosynthesis pathway (Ljung, 2013; Kasahara, 2016; Casanova‐Sáez & Voss, 2019) dominates. This is initiated by the conversion of tryptophan to IPyA by TAA1‐related enzymes (TARs), while YUCCA‐related enzymes (YUCs) convert IPyA to the active auxin IAA (Stepanova *et al*., 2008; Tao *et al*., 2008; Mashiguchi *et al*., 2011; Won *et al*., 2011). The bryophyte genomes encode homologous sequences, and OE of an *M. polymorpha YUC* homolog results in elevated IAA production, and morphological changes similar to those induced by exogenous IAA treatment were found in both *MpTAR* and *MpYUC* OE lines (Eklund *et al*., 2015).

Thus, to widen our understanding of auxin biosynthesis in early land plant evolution and to establish tools to study spatiotemporal changes in auxin sensing and responses, we investigated the IPyA pathway in *P. patens*. Representative *PpYUC* and *PpTAR* homologs were selected for OE and knock‐out (KO) studies. This revealed that the IPyA pathway is the major *P. patens* auxin biosynthesis route controlled by largely redundant gene copies, and that the first step is rate limiting. As the genetic redundancy was less prominent in the reproductive organs, the KO lines allowed us to use the initiation and development of gametangia to study the role of auxin in stem cells, as well as in organ and gamete differentiation. Apart from following the effect of reduced auxin production on reproductive development, we also compared the sites of auxin biosynthesis and sensing. We used *PpTAR* and *PpYUC* transcriptional reporters as well as the PpR2D2 reporter for AuxRE‐ and ARF‐independent detection of auxin sensing activity (Thelander *et al*., 2019). These studies revealed that vegetative as well as reproductive gametophore stem cells produce auxin, but that this auxin is not sensed by the stem cells. Instead, auxin sensing increases in daughter cells, aiding in the control of cell division, expansion and thus in the final differentiation/morphogenesis. Early egg and sperm precursor cells are dependent on a certain degree of auxin sensing, while the final differentiation of the gametes appears to be a low auxin sensing process.

## Materials and Methods

### Plant material, growth conditions, moss transformation and crosses

The *Physcomitrium* (*Physcomitrella*) *patens* ecotype Reute was used as WT and background to all transgenic lines produced (Hiss *et al*., 2017). The following lines were published previously: *PpR2D2‐2* and *PpR2D2‐3* (Thelander *et al*., 2019), *PpPINBpro::GFP‐1*, *PpPINCpro::GFP‐1*, *PpPINDpro::GFP‐1* and *PpPINApro::PpPINA‐GFP‐2* (Viaene *et al*., 2014, Gransden ecotype). For cultivation, spores were germinated, or protonemal tissue was subcultivated, on MM plates containing BCD medium (Thelander *et al*, 2007) supplemented with 5 mM ammonium tartrate (Sigma Aldrich A2956) and 0.8% agar (Sigma Aldrich A1296). The protonemal tissue was shaped into 2 mm balls, inoculated on solid BCD medium in 25 mm deep Petri dishes (VWR International PHOE305; Radnor, PA, USA) and grown at 25°C under constant white light from F25T8/TL741 fluorescent tubes (Philips, Somerset, NJ, USA) at 35 µmol m^−2^ s^−1^ in a Percival Scientific CU‐41L4 growth chamber (Perry, IA, USA) for 5–6 wk. To induce reproductive organ formation the plates were then transferred to a Sanyo MLR‐350 light chamber with 8 h of light (30 µmol m^−2^ s^−1^) per day at 15°C. Protoplast transformation and crosses were carried out as previously described (Schaefer *et al*., 1991; Thelander *et al*., 2019). Stable transformants and higher order mutants were selected in the presence of 50 µg ml^−1^ hygromycin (Duchefa H0192; Haarlem, the Netherlands), G418 (11811023; Thermo Fisher Scientific, Waltham, MA, USA) or zeocin (R250‐01; Thermo Fisher Scientific).

### IAA metabolite measurements

Protonemal tissue, blended and re‐grown twice, was grown for 9 d on cellophane (A.A. Packaging Limited, Preston, UK) overlayed MM plates in continuous light, and then harvested, weighed and frozen in liquid nitrogen. At least five independent biological replicates were harvested for the *PpTARA OE*, *PpTARF OE*, *PpYUCA OE*, *PpYUCC OE*, *Pptara‐1*, *Pptarb‐1* and *Pptaratarb‐1* lines and 20 biological samples for WT. The extraction, purification and the LC‐MS analysis of endogenous IAA, its precursors and metabolites were carried out according to Novák *et al*. (2012). Briefly, *c*. 25 mg of frozen material per sample was homogenized using a bead mill (27 Hz, 10 min, 4°C; MixerMill, Retsch GmbH, Haan, Germany) and extracted in 1 ml of 50 mM sodium phosphate buffer containing 1% sodium diethyldithiocarbamate and a mixture of 13C_6_‐ or deuterium‐labeled internal standards. After centrifugation (23 000 *g*, 15 min, 4°C), the supernatant was divided into two aliquots: the first aliquot was derivatized using cysteamine (0.25 M; pH 8; 1 h; room temperature; Sigma‐Aldrich), and the second aliquot was immediately further processed as follows. The pH of the sample was adjusted to 2.5 by 1 M HCl and applied on preconditioned solid‐phase extraction columns (30 mg 1 ml, Oasis HLB; Waters Inc., Milford, MA, USA). After sample application, the column was rinsed with 2 ml 5% methanol. Compounds of interest were then eluted with 2 ml 80% methanol. The derivatized fraction was purified alike. MS analysis and quantification were performed by using an LC‐MS/MS system comprising a 1290 Infinity Binary LC System coupled to a 6490 Triple Quad LC/MS System with Jet Stream and Dual Ion Funnel technologies (Agilent Technologies, Santa Clara, CA, USA).

### Gene inventory and phylogenetic reconstruction

The full complement of *TAR* and *YUC* genes in the genome sequences of Arabidopsis (TAIR 10) and *P. patens* (v.3.3) were identified by Blast searches against the Phytozome database (v.12.1.5) and protein sequences deduced from primary gene models were retrieved (AtTAA1: AT1G70560.1; AtTAR1: AT1G23320.1; AtTAR2: AT4G24670.1; AtTAR3: AT1G34040.1; AtTAR4: AT1G34060.1; AtYUC1: AT4G32540.1; AtYUC2: AT4G13260.1; AtYUC3: AT1G04610.1; AtYUC4: AT5G11320.1; AtYUC5: AT5G43890.1; AtYUC6: AT5G25620.2; AtYUC7: AT2G33230.1; AtYUC8: AT4G28720.1; AtYUC9: AT1G04180.1; AtYUC10: AT1G48910.1; AtYUC11: AT1G21430.1; PpTARA: Pp3c21_15370V3.1; PpTARB: Pp3c18_15140V3.1; PpTARC: Pp3c17_6500V3.1; PpTARD: Pp3c26_12520V3.1; PpTARE: Pp3c25_6670V3.1; PpTARF: Pp3c5_24670V3.1; PpYUCA: Pp3c3_18590V3.1; PpYUCB: Pp3c11_11790V3.1; PpYUCC: Pp3c1_11500V3.1; PpYUCD: Pp3c2_27740V3.1; PpYUCE: Pp3c13_21970V3.1; PpYUCF: Pp3c3_20490V3.1). Phylogenetic reconstructions were produced with the Megax software (v.10.1.5; Kumar *et al*., 2018): protein alignments produced with the muscle algorithm (default settings) were used to construct trees with the maximum likelihood method (default settings) and 500 replications of bootstrapping.

### Construct building and transgenic lines

The generation of new transgenic lines is described in Supporting Information Methods S1–S4, and their molecular verification is shown in Figs S1–S6. For lists of constructs and primers, see Tables S1 and S2, respectively.

### RT‐qPCR

Reverse transcriptase quantitative PCR (RT‐qPCR) determination of *PpTAR* transcript abundance is described in Methods S5.

### Mutant phenotyping

Colonies and vegetative shoots were documented using a Leica M205 FA stereo microscope and Las af software (Leica Microsystems, Wetzlar, Germany). Colony diameters were measured in imagej (Schneider *et al*., 2012). To analyze reproductive organ development, entire shoot apices were harvested at indicated time points and all leaves were detached under a stereo microscope (Leica MZ16; Leica Biosystems, Heidelberg, Germany) leaving the reproductive organs exposed at the apex. The shoot apices were mounted on objective glasses in 30% glycerol and reproductive organs were analyzed using a Leica DMI4000B microscope with differential interference contrast (DIC; Nomarski) optics, a Leica DFC360FX camera, and the Las af (Leica Microsystems) software. Adobe Photoshop CC was used to adjust intensity and contrast, remove background, mark borders and cells, and to visualize entire late‐stage archegonia by merging two to three images taken at 63× or 100× magnification. All presented experiments were performed in at least two independent biological data sets. Microsoft Excel was used to create bar charts, calculate means and SD, and to perform a Student’s *t*‐test where indicated in the Results section.

### Confocal microscopy

For fluorescence reporter signal analysis, shoot apices were harvested as for phenotypic analysis, mounted in water and immediately detected with an inverted Zeiss 780 confocal microscope at 20× (Plan‐Apochromat, NA 0.8) or 63× (C‐Apochromat, water immersion, NA 1.20) magnification. Excitation/detection parameters were 488 nm/491–598 nm for green fluorescent protein (GFP) and 633/647–721 nm for Chl auto‐fluorescence. Both *PpTARA::GFP‐GUS* lines analyzed had the same signal strength in all analyzed developmental stages and organs, and selected pictures of both are shown in the figures. *PpPINB*, *PpPINC* and *PpPIND* GFP reporter signals were detected with an inverted Zeiss 800 confocal microscope at 40× and 63× magnification. Excitation/detection parameters were 488 nm/410–617 nm for GFP and 655/656–721 nm for Chl auto‐fluorescence. All images are snapshots of a single focal plane with selected channels overlaid using the Zen black software.

PpR2D2 output was detected and quantified as previously described with the addition that late‐stage archegonia were analyzed with the 20× objective while all other organs were analyzed with the 63× objective (Thelander *et al*., 2019). PpR2D2 output (images as well as signal quantifications) presented within a figure is always directly comparable, but this is not necessarily the case between figures due to differences in microscopy settings and/or image processing.

## Results

### The IPyA pathway is dominant in *P. patens* and manipulation of *TAR* and *YUC* expression drastically affects auxin levels

To investigate if the IPyA pathway is important for auxin biosynthesis in *P. patens*, we measured IAA metabolite levels in chloronema‐enriched protonemal tissue. Of the four IAA precursors representing different potential routes from tryptophan (TRP) to IAA, the level of IPyA is high, and tryptamine (TRA) low, while indole‐3‐acetonitrile (IAN) and indole‐3‐acetamide (IAM) are nondetectable, establishing IPyA as the dominant IAA precursor in *P. patens* (Table 1). Also, the amounts of the oxidative catabolites oxIAA and oxIAA‐glucose are high, of amino acid conjugates extremely low, and of glucose conjugates significant, showing that auxin conjugation occurs in *P. patens*. Overall, the metabolite profile is similar to that of flowering plants (Novák *et al*, 2012).

**Table 1:** Auxin metabolite profile in *Physcomitrium (Physcomitrella) patens* chloronema‐enriched protonema.

Abbreviation	Compound	Concentration (pmol g^−1^ FW; mean ± SD)
TRP	Tryptophan	21 785 ± 5712
TRA (TAM)	Tryptamine	11.77 ± 3.56
IAN	Indole‐3‐acetonitrile	UDL
IAM	Indole‐3‐acetamide	UDL
IPyA	Indole‐3‐pyruvic acid	82.99 ± 17.22
IAA	Indole‐3‐acetic acid	8.50 ± 1.75
oxIAA	2‐Oxoindole‐3‐acetic acid	27.17 ± 5.16
oxIAA‐Glc	oxIAA‐glucose	46.69 ± 12.41
IAA‐Glc	IAA‐glucose	7.84 ± 2.08
IAA‐Asp	IAA‐aspartate	0.28 ± 0.09
IAA‐Gly	IAA‐glycin	0.58 ± 0.24
IAA‐Glu	IAA‐glutamate	0.62 ± 0.15
IAA‐Val	IAA‐valine	UDL
IAA‐Leu	IAA‐leucine	0.08 ± 0.03
IAA‐Phe	IAA‐phenylalanine	UDL
IAA‐Trp	IAA‐tryptophane	UDL

FW, fresh weight; UDL, under detection limit.

**Fig. 1:**
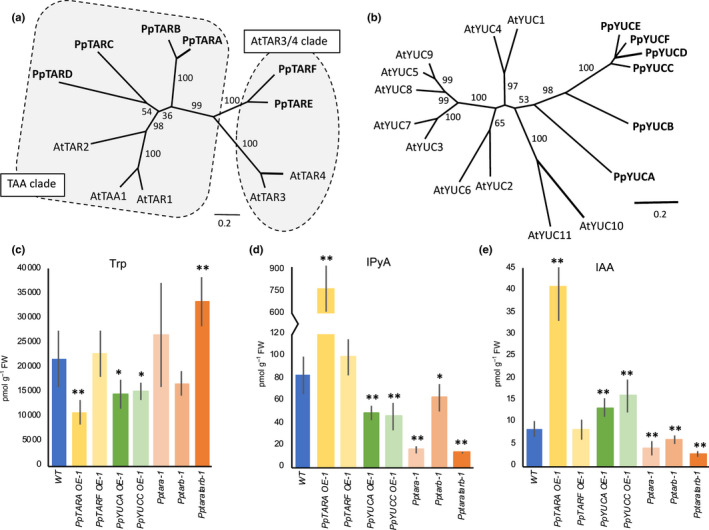
*TAR* and *YUC* multigene families promote auxin biosynthesis in *Physcomitrium (Physcomitrella) patens*. (a, b) Unrooted phylogenetic trees produced by the maximum likelihood method to infer evolutionary relationship of (a) TAR and (b) YUC proteins encoded by Arabidopsis (At) and *P. paten*s (Pp) genes. Branch lengths indicate genetic distance and numbers on branches are bootstrap values (only values above 50% are shown). The TAR proteins fall in two distinct clusters referred to as the TAA and the AtTAR3/4 clades, respectively. (c–e) Level of (c) tryptophan (Trp), (d) indole‐3‐pyruvic acid (IPyA) and (e) IAA in protonemal tissue of *P. patens* wild type (WT) and stated over‐expressor (OE) and knock‐out mutant lines. Error bars represent the SD of the mean of at least five biological replicates. Asterisks indicate statistically significant differences compared to WT: *, *P* < 0.05; **, *P* < 0.01 (Student’s *t*‐test). See also Supporting Information Table S3.

In accordance with previous inventories and comprehensive phylogenetic reconstructions, we identified multiple tryptophan aminotransferase (*TAR)* and *YUCCA* (*YUC*) genes in *P. patens* (Yue *et al*., 2014; Eklund *et al*., 2015; Poulet & Kriechbaumer, 2017; Romani, 2017). The genome possesses six *TAR* homologs on separate chromosomes which fall in two distinct phylogenetic groups (Fig. 1a). *PpTARA–D* cluster with the Arabidopsis genes *AtTAA1*, *AtTAR1* and *AtTAR2* demonstrated to play a role in auxin biosynthesis (TAA clade in Fig. 1a; Stepanova *et al*., 2008; Tao *et al*., 2008). *PpTARE–F* cluster with *AtTAR3* and *AtTAR4* (AtTAR3/4 clade in Fig. 1a) which have been proposed to encode alliinases rather than tryptophan aminotransferases, but a functional characterization is pending (Turnaev *et al*., 2015). The intron positions within the coding region of all Arabidopsis and *P. patens TAR* homologs is conserved, suggesting a common descent for all members (Fig. S7).

Of the six *YUC* homologs, *PpYUCA* and *PpYUCB* share an identical exon–intron organization with the majority of the 11 Arabidopsis *YUC* genes while the four highly similar genes *PpYUCC–F* lack introns (Figs 1b, S8). With the exception of *PpYUCA* and *PpYUCF*, which both reside on chromosome 3, the genes are found on separate chromosomes. Phylogenetic analysis indicates that all *PpYUC* genes are more similar to each other than to any Arabidopsis gene and, even if the support is low, that their closest Arabidopsis relatives may be *AtYUC10* and *AtYUC11* as previously reported (Fig. 1b; Poulet & Kriechbaumer, 2017).

Next, we generated OE lines of *PpTARA*, *PpTARF*, *PpYUCA* and *PpYUCC* to represent the main branches in the TAR and YUC trees (Fig. 1a,b). Two lines of each construct exhibiting high transgene expression in protonema were selected for IAA metabolite profiling (Figs 1c–e, S1; Table S3). As expected, IPyA levels were strongly elevated upon *PpTARA* OE and significantly reduced by *PpYUCA* and *PpYUCC* OE, showing that PpTAR enzymes catalyze the production of IPyA while PpYUC activity consumes it (Fig. 1d). Accordingly, the IAA level is higher in both *PpYUC* OEs compared to WT, and even further so in *PpTARA* OE, indicating that PpTAR, but not PpYUC enzymes, represent the major rate‐limiting step in *P. patens* IAA biosynthesis (Fig. 1e). *PpTARF* OE does not influence the level of any measured auxin metabolite (Fig. 1c–e; Table S3), suggesting that *PpTARF* and *PpTARE* are not involved in IAA biosynthesis.

We also created KO mutants for the four *AtTAA1*‐like *PpTAR* genes and all six *PpYUC* genes. None of the *Ppyuc* single mutants showed a significant protonemal phenotype, indicating a high degree of *PpYUC* redundancy, while at least one *PpTAR* single mutant, *Pptara*, was significantly affected in colony diameter (Fig. S9). In line with this, both published trascriptome data (ecotype Gransden) and our qPCR analysis (ecotype Reute) show that *PpTARA* is the most highly expressed *PpTAR* in chloronema, followed by *PpTARB* and *PpTARC*, while *PpTARD* expression is negligible (Ortiz‐Ramirez *et al*., 2016, Fig. S10). Based on this, we created *Pptaratarb* double KO mutants and measured the levels of IAA and IAA metabolites in *Pptara*, *Pptarb* and *Pptaratarb*. Consistent with a role in IPyA‐dependent IAA biosynthesis, the level of both IPyA and IAA were reduced slightly in the WT‐resembling *Pptarb*, strongly in *Pptara* and even further in *Pptaratarb* compared to WT (Fig. 1d,e).

### Auxin is synthesized but not sensed in shoot apical stem cells and its immediate cleavage products

**Fig. 2:**
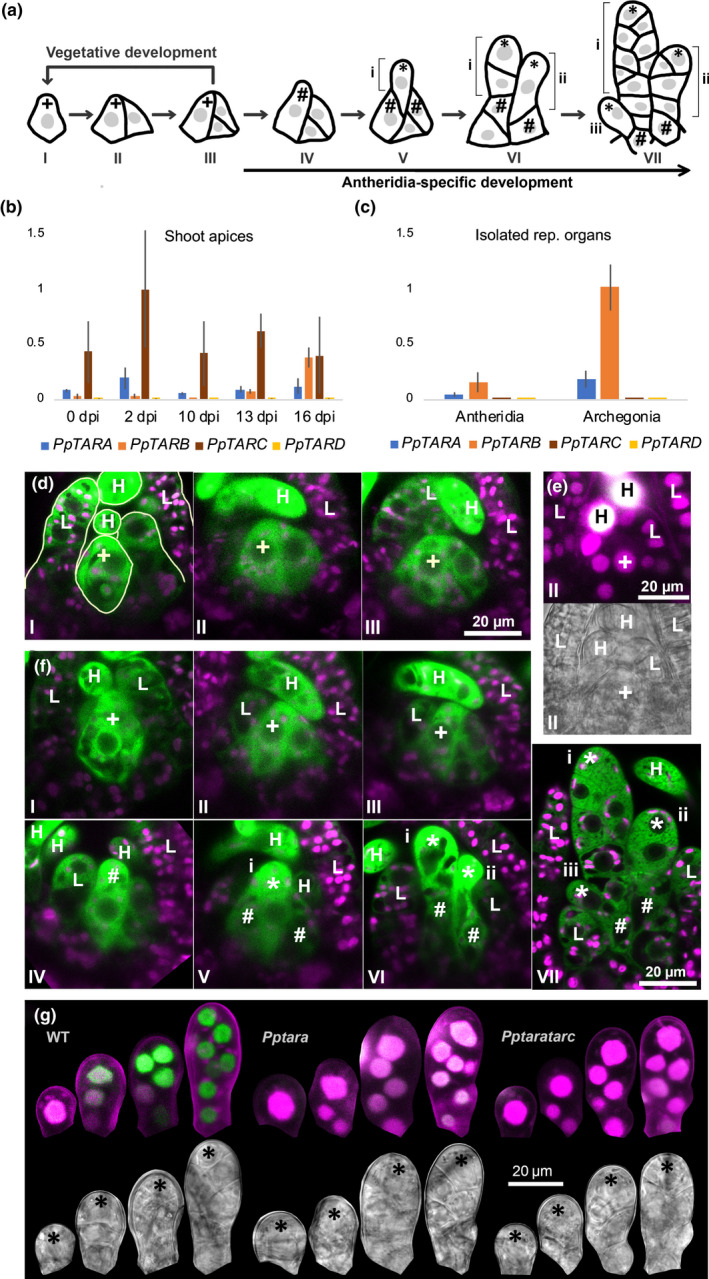
*PpTAR* expression and auxin sensing during transition from vegetative to early male reproductive development in *Physcomitrium (Physcomitrella) patens*. (a) Schematic drawing of cell divisions in the shoot apical stem cell region during the transition from vegetative to early male reproductive development. (b, c) Relative *PpTAR* transcript abundance assayed by qPCR in (b) detached shoot apices harvested at different time points after transfer to inductive conditions (10 dpi apices possess young antheridia; 13 dpi apices possess mid‐stage antheridia and young archegonia; 16 dpi apices possess mature antheridia and mid‐stage archegonia) and (c) in isolated antheridia and archegonia bundles. Each data point represents an average of three independent biological replicates and error bars indicate SD. (d) *PpTARA::GFPGUS‐1/2* reporter output in the stem cell region of vegetative shoot apices before the transfer to inductive conditions. (e) Auxin sensing visualized by PpR2D2‐3 output in the stem cell region of a WT vegetative shoot apex before the transfer to inductive conditions. (f) *PpTARA::GFPGUS‐1/2* reporter output in shoot apices in different stages of early male reproductive development. (g) Auxin sensing visualized by PpR2D2‐3 output in one‐ to eight‐celled antheridia initials in WT, *Pptara* (*PpR2D2‐3 Pptara‐1*) and *Pptaratarc* (*PpR2D2‐3 Pptaratarc‐1*) backgrounds. For related signal quantification data, see Fig. S12. In (d, f), merged images of confocal channels detecting green fluorescent protein (green) and chloroplast autofluorescence (magenta) are shown. In (e, g), a maximum‐intensity projection of a Z‐stack with confocal channels detecting mDII‐nVENUS (green) and DII‐nTdTOMATO (magenta) merged (upper) and a differential interference contrast image from a selected Z‐plane (lower) are shown for each item. Microscopy settings and image processing are identical between the different items. A high green : magenta signal ratio indicates high auxin sensing (for details, see Thelander *et al*., 2019). Key to symbols: +, vegetative shoot apical stem cell; #, antheridium initial stem cell; *, antheridium apical stem cell; I–VII, consecutive developmental stages; i–iii, consecutive antheridia initials; L, leaf initial; H, hair.

Shoot growth in *P. patens* is sustained by a single apical stem cell which cleaves off daughter cells (Fig. 2a) contributing to stem growth, lateral leaves and hairs (Harrison *et al*., 2009; Kofuji *et al*., 2018). qPCR suggests that at least *PpTARA* and *PpTARC* are expressed in detached vegetative shoot apices (Fig. 2b). We thus generated and analyzed *PpTAR* transcriptional reporters as a proxy for where and when auxin biosynthesis takes place in this region. *PpTARA* expression is relatively strong in the apical stem cells of vegetative adult shoots and somewhat weaker in its most immediate cleavage products (Fig. 2d), while no consistent *PpTARC* expression could be observed in the stem cell region (data not shown). As the *PpTARB* and *PpTARD* reporters failed to detect any expression in the stem cell region or in reproductive organs, they are not further discussed (data not shown). Mapping of auxin sensing in the stem cell region is challenging because promoter‐based reporters (GmGH3, DR5, DR5revV2) are not informative and the ratiometric PpR2D2 reporter is expressed at low levels in these cells (Thelander *et al*., 2019). Still, the PpR2D2 reporter clearly shows that all cells in the stem cell region display extremely low auxin sensing (Fig. 2e). As *Pptaratarb* and *Pptaratarc* double mutants produce functional shoots (data not shown), although the *Pptaratarc* stem is dwarfed (Fig. S11), the shoot apical stem cell appears unaffected by the reduced auxin biosynthesis. This indicates that the shoot apical stem cell and its immediate cleavage products represent a hotspot for auxin biosynthesis but neither sense the auxin nor depend on it for their function.

### Auxin biosynthesis appears dispensable for the primary initiation of reproductive development

The *P. patens* shoot apex transits from a vegetative to a monoecious reproductive program in response to low temperature and short day‐length (Hohe *et al*., 2002; Landberg *et al*., 2013; Hiss *et al*., 2017; Kofuji *et al*., 2018). It first triggers the initiation of male antheridia by imposing changes in the behavior of the shoot apical stem cell and its most recent daughter cell, both of which take on new identities as antheridium initial stem cells (Fig. 2a; Kofuji *et al*., 2018). Unlike other described *P. patens* stem cell types, antheridium initial stem cells cleave off daughter cells distally rather than proximally, which immediately acquire identities as antheridium apical stem cells driving antheridia development (Fig. 2a; Kofuji *et al*., 2018). The two antheridium initial stem cells cleave off multiple antheridium apical stem cells in an alternating manner so that the most recent one is positioned outside its predecessors to eventually give rise to bundles with the oldest organ in the middle (Landberg *et al*., 2013; Kofuji *et al*., 2018).

Sensing of the environmental conditions inducing the transition to reproductive development appears to be independent of auxin biosynthesis. The first transitional sign, the emergence of a protrusion from the apical stem cell which marks the switch from proximal to distal cell division, occurs with similar timing in WT and *PpTAR* mutants (data not shown). We were also unable to score significant changes of *PpTAR* transcript levels, or in the output from the *PpTARA* and PpR2D2 reporters, in the stem cell region of shoots before or during the transit process (Fig. 2b,d,f; data not shown).

### A gradual increase in *PpTAR*‐dependent auxin sensing is needed for early antheridia development

The newly established antheridium apical stem cell cleaves off daughter cells proximally to form a stage‐2 antheridium consisting of about eight cells arranged in two cell files (Fig. 2a VII; stages here and below according to Landberg *et al*., 2013; Kofuji *et al*., 2009). As *PpTARA‐C* transcripts could be detected in developing reproductive organs by qPCR (Fig. 2b,c), we used our transgenic tools to estimate their role in organ pattern formation. *PpTARA* is expressed uniformly in stage‐2 organs at levels higher than in the antheridium initial stem cell from which it originates (Fig. 2f). PpR2D2 auxin sensing also increases uniformly in one‐ to three‐celled organs, while further increase is restricted to the apical part in four‐ to eight‐celled organs resulting in a gradient (Figs 2g, S12). This gradual increase in auxin sensing is dependent on auxin biosynthesis as PpR2D2 readout is significantly reduced in *Pptara* and *Pptaratarc* young antheridia (Figs 2g, S12).

**Fig. 3:**
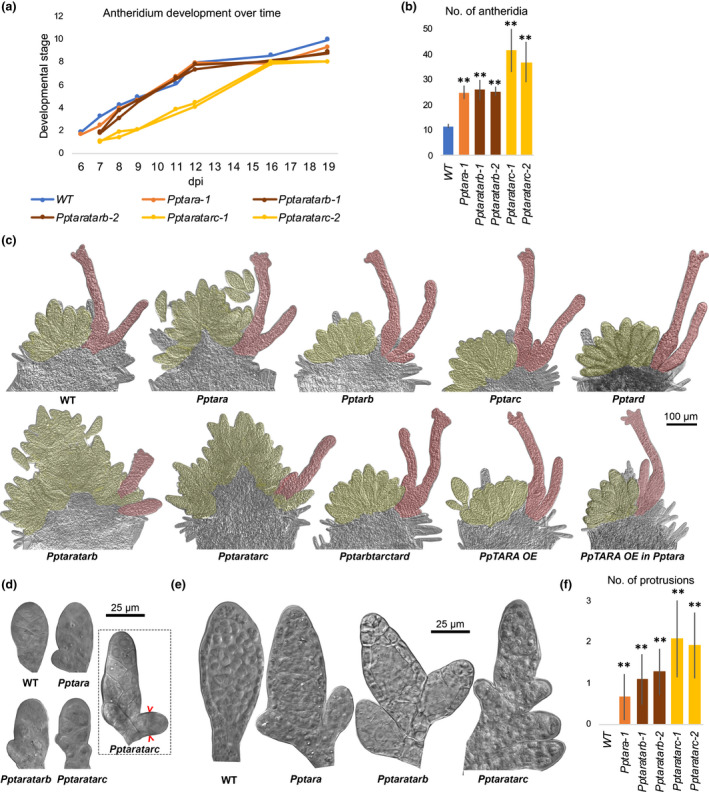
*Pptar* mutant phenotype relating to the initiation and early development of *Physcomitrium (Physcomitrella) patens* antheridia. (a) Graph showing the average developmental stage (Landberg *et al*., 2013) of the most advanced antheridium in shoot apices of selected *Pptar* mutant lines vs WT at different time points after the transfer to inductive conditions (dpi). The two *Pptaratarc* lines differ significantly (*P* < 0.01) from the WT at both 8 and 12 dpi as determined in a Dunnett’s test performed using the R package multicomp (Dunnett, 1955; Hothorn *et al*., 2008). (b) Bar graph showing the average number of antheridia per bundle when the most advanced antheridium has reached stage 8 in selected *Pptar* mutant lines vs WT. (c) Mature reproductive shoot apices from *Pptar* mutants and the WT demonstrating the hyperformation of antheridia and stunted archegonia in *Pptara*, *Pptaratarb* and *Pptaratarc*. Also note that *PpTARA OE* restores the *Pptara* phenotype while no phenotype is caused in WT (see also Supporting Information Fig. S13). Antheridia and archegonia have been false shaded in yellow and red, respectively, for clarity. (d) Representative stage 2 antheridia from the WT and selected *Pptar* mutants. Note outgrowths from the base of mutant organs which eventually will result in ectopic antheridia. The dashed square shows a somewhat later *Pptaratarc* organ demonstrating that the first division undertaken by ectopic outgrowths is periclinal (marked by red brackets). (e) Representative late‐stage antheridia from the WT and selected *Pptar* mutants. Note ectopic antheridia outgrowth from the base of mutant organs. (f) Bar graph showing the average frequency of ectopic outgrowths from the basal parts of late‐stage antheridia from WT and selected *Pptar* mutants. In (b, f), error bars indicate SD and double asterisks indicate a statistically significant difference from the WT (Student’s *t*‐test: **, *P* < 0.01).

The lack of increase in auxin sensing in young *Pptaratarc* antheridia delays the transition from stage 1 to stage 2 by 2–3 d, but the organ then goes through subsequent developmental stages at normal pace (Fig. 3a). This may be the result of a delay in the formation of the antheridium apical stem cell from the antheridium initial stem cell, and/or a delay in the first cell division executed by the newly formed antheridium apical stem cell. The delay in the stage 1 to 2 transition is seen mainly in *Pptaratarc*, indicating that both *PpTARA* and *PpTARC* contribute to auxin biosynthesis at this stage (Fig. 3a). In line with this, auxin sensing is even lower in *Pptaratarc* than in *Pptara* early during stage 2 development (Figs 2g, S12). This indicates that the establishment and/or activity of the antheridium apical stem cell requires a minimal *PpTAR*‐dependent auxin activity.

**Fig. 4:**
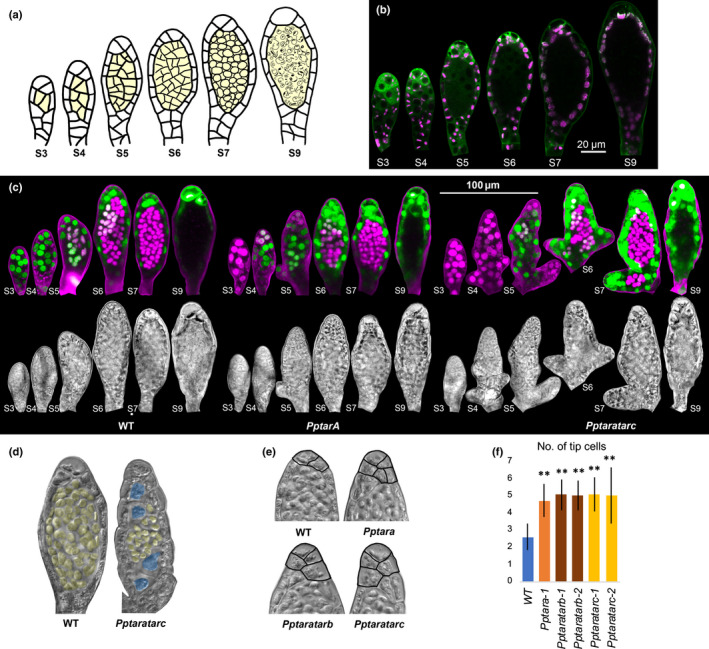
*PpTAR* expression, auxin sensing and *Pptar* mutant phenotype in stage 3–9 *Physcomitrium (Physcomitrella) patens* antheridia. (a) Schematic drawing of stage 3–9 antheridia development. Inner spermatogenous cells are shaded in yellow. (b) *PpTARA::GFPGUS‐1/2* reporter output in stage 3–9 antheridia. For each stage, a merged image of confocal channels detecting green fluorescent protein (green) and chloroplast autofluorescence (magenta) is shown. (c) Auxin sensing visualized by PpR2D2‐3 output in stage 3–9 antheridia from WT, *Pptara* (*PpR2D2‐3 Pptara‐1*) and *Pptaratarc* (*PpR2D2‐3 Pptaratarc‐1*) backgrounds. For each organ a maximum‐intensity projection of a Z‐stack with confocal channels detecting mDII‐nVENUS (green) and DII‐nTdTOMATO (magenta) merged (upper) and a differential interference contrast image from a selected Z‐plane (lower) are shown. Microscopy settings and image processing are identical between the different organs. A high green : magenta signal ratio indicates high auxin sensing (for details, see Thelander *et al*., 2019). For related signal quantification data, see Supporting Information Fig. S14. (d) Stage 7 antheridia from WT and the *Pptaratarc* mutant demonstrating that a subset of mutant inner cells fail to proliferate (shaded in blue). Spermatogeneous inner cells have been shaded in yellow. See also Fig. S15. (e) Upper half of stage 7 antheridia from WT and selected *Pptar* mutants demonstrating ectopic tip cells in the latter. Borders of cells classified as tip cells have been marked for clarity. (f) Bar graph showing the average number of tip cells per stage 8 antheridium in selected *Pptar* mutants vs WT. Error bars indicate standard deviation and double asterisks indicate a statistically significant difference from the WT (Student’s *t*‐test: **, *P* < 0.01).

In addition, the number of antheridia per bundle is significantly increased in *Pptara* and *Pptaratarb*, and even further so in *Pptaratarc* (Fig. 3b,c). This and all other phenotypes discussed below require that *PpTARA* has been deleted and they can all be at least partially restored by *PpTARA* OE, while the *Pptarb*, *Pptarc, Pptard* and *Pptarbtarctard* mutants produce functional reproductive organs indistinguishable from WT (Figs 3c, S13). *PpTARA* thus appears particularly important for reproductive development while *PpTARB* and *PpTARC* contribute, at least in the absence of *PpTARA*. Despite several attempts, we have not been able to generate *Pptaratarbtarc* triple KO lines, suggesting that these three genes together provide an essential function (data not shown). Antheridia hyperformation is due to ectopic outgrowth of extra organs from basal cells of pre‐existing stage‐2 antheridia, which form a protrusion and undergo a periclinal division (Fig. 3d). The ectopic cell formed acquires the identity of an antheridium apical stem cell which cleaves off cells proximally in two adjacent cell files to form an early ectopic antheridium which develops more or less normally and show the expected PpR2D2 auxin sensing at their tip and jacket cells (Fig. 4c). The ectopic antheridia can either be initiated by basal cells forming the stalk, making them appear as stand‐alone organs, or by basal cells on the body part, resulting in antheridia that appear branched, a phenomenon that is never seen in WT but is frequent in *Pptara* and even more so in *Pptaratarb* and *Pptaratarc* (Fig. 3e,f). The basal part of the antheridium shows significantly lower auxin sensing in *Pptara*, and even more so in *Pptaratarc*, compared to WT (Figs 2g, S12). This indicates that *PpTAR*‐dependent auxin sensing in basal cells of stage‐2 antheridia suppress them from undergoing periclinal/distal divisions to produce antheridium apical stem cells, a task normally carried out only by antheridium initial stem cells.

### *PpTAR*‐dependent auxin sensing controls cell division activity in the antheridium apex and spermatogenesis is associated with minimal auxin sensing

At the start of stage 3, subapical antheridia cells undergo periclinal divisions to set off a few inner cells (Fig. 4a). Simultaneously, the uniform *PpTARA* expression from stage 2 changes to a gradient with an apical maximum at stages 3 and 4, while the auxin sensing gradient is partly evened out as sensing in the basal parts increases (Figs 4b,c, S14). The new inner cells initially share *PpTARA* expression and auxin sensing levels with the subapical jacket cell from which they were derived (Figs 4b,c, S14). The inner cells further divide to produce a number of considerably smaller spermatogenous cell initials, each of which go through spermatogenesis during stages 7–9 to form a biflagellated coiled sperm (Fig. 4a; Landberg *et al*., 2013). While inner cells form normally in all *Pptar* mutants up until stage 4, a subset of these cells fails to divide in *Pptaratarc* and never enter the spermatogenesis program (Figs 4d, S15). Auxin sensing is severely reduced in the inner stage 3–4 cells, suggesting that further proliferation of stage 4 inner cells, and thus the production of normal sperm counts, requires a minimal level of auxin sensing which is not always met in *Pptaratarc* but is apparently satisfied in the *Pptara* single mutant (Figs 4c, S14).

From stage 5, both *PpTARA* expression and auxin sensing in inner cells starts to decrease gradually to reach extremely low levels at around the onset of spermatogenesis in stage 7 (Fig. 4b,c). Spermatogenous cells from all *Pptar* mutants, including the *Pptaratarc* cells that have entered cell division, are able to complete the spermatogenesis program. Sperm from the two double mutants were able to fertilize egg cells of the male sterile ecotype Gransden, resulting in the production of kanamycin‐resistant heterozygous sporophytes, although at very low frequency. These data suggest that final sperm differentiation is a low‐auxin sensing process.

In parallel with the development of the inner cells, cells in the unicellular jacket layer undergo anticlinal divisions to keep up with organ growth (Fig. 4a). In WT, *PpTARA* expression in the outer cell layer becomes largely restricted to the extreme tip cells at around stage 6 before it is completely lost before organ opening (Fig. 4b). Auxin sensing remains high and relatively uniform in the tip cells and in other outer cells until organ opening (Figs 4c, S14). As expected, auxin sensing is dramatically reduced in the tip cells of stage 6 *Pptara* and *Pptaratarc* antheridia and this correlates with the formation of extra cells at the antheridia tips (Fig. 4e,f). This indicates that in comparison to WT the mutant antheridium apical stem cell remains active for longer and cleaves off more daughter cells after the first inner cell has formed. *PpTAR*‐dependent auxin sensing above a certain level may therefore be needed to cease cell division activity of the antheridium apical stem cell.

### *PpTAR* expression marks the *de novo* establishment of a female archegonia bundle at an independent lateral position

**Fig. 5:**
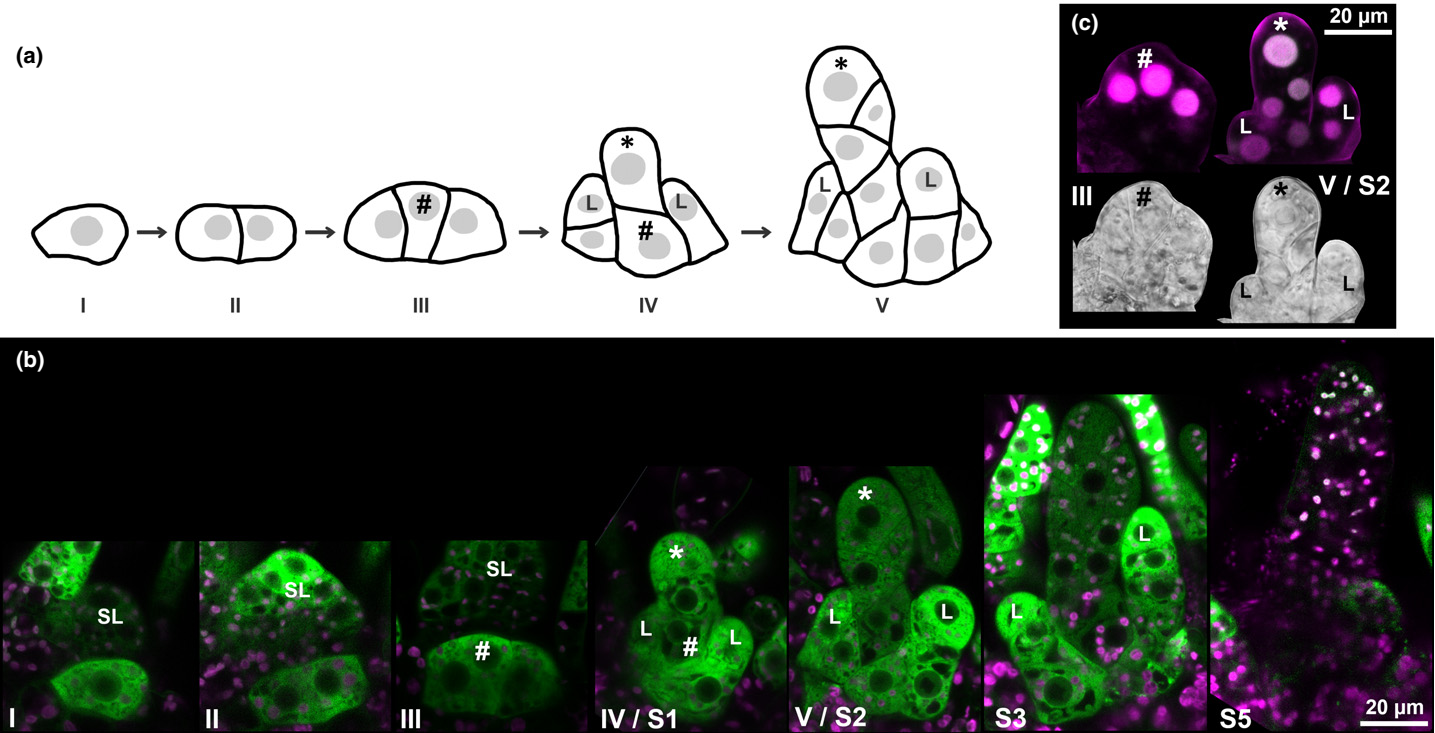
*PpTAR* expression and auxin sensing during archegonia initiation in *Physcomitrium (Physcomitrella) patens*. (a) Schematic drawing of cell divisions during the *de novo* establishment of a stem cell region producing archegonia. (b) *PpTARC::GFPGUS‐1* reporter output during early archegonia initiation. For each stage, a merged image of confocal channels detecting green fluorescent protein (green) and chloroplast autofluorescence (magenta) is shown. (c) Auxin sensing during early archegonia initiation as visualized by PpR2D2‐3 output. Both a differential interference contrast image from a selected Z‐plane (lower) and a maximum‐intensity projection of a Z‐stack with confocal channels detecting mDII‐nVENUS (green) and DII‐nTdTOMATO (magenta) merged (upper) are shown. A high green : magenta signal ratio indicates high auxin sensing (for details, see Thelander *et al*., 2019). Key to symbols: #, probable archegonium initial stem cell; *, archegonium apical stem cell; I–V, consecutive developmental stages; L, leaf initial; SL, leaf separating antheridia and archegonia bundle; S1/2/3/5, archegonia stages according to Landberg *et al*. (2013).

**Fig. 6:**
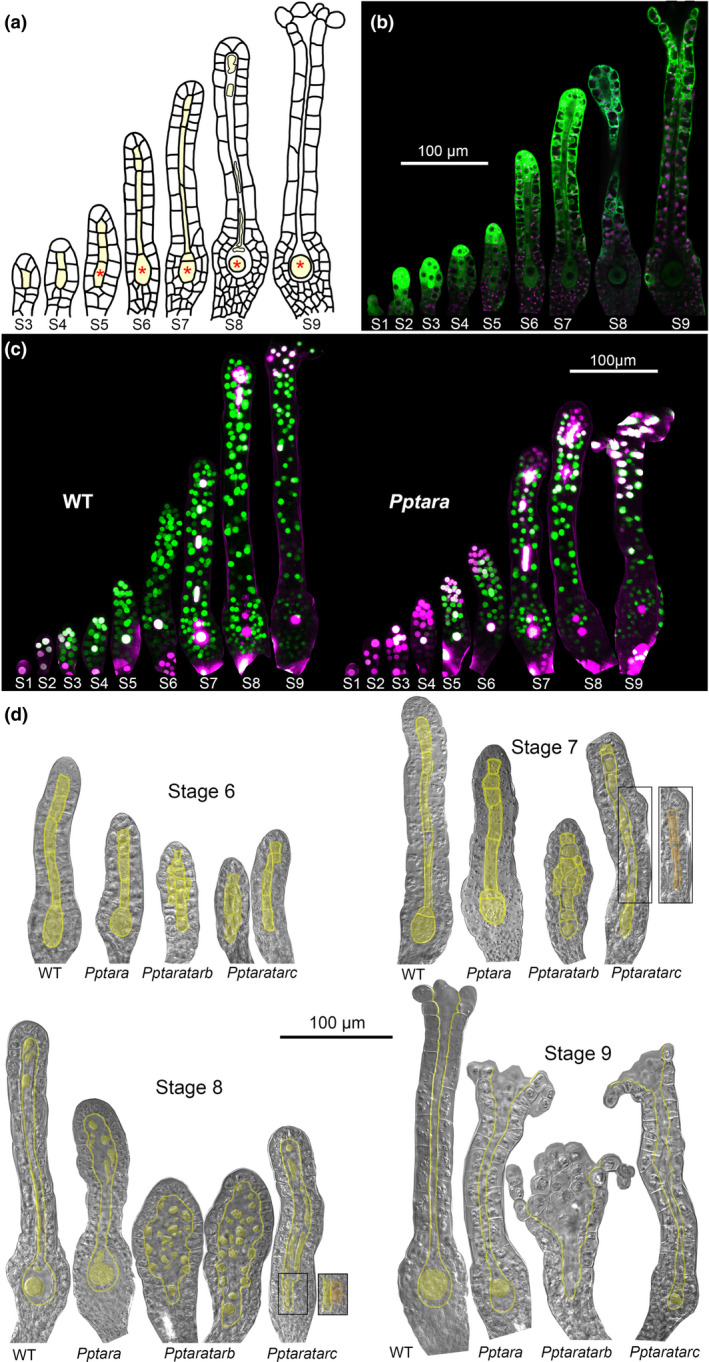
*PpTARA* expression, auxin sensing and *Pptar* mutant phenotype in *Physcomitrium (Physcomitrella) patens* archegonia. (a) Schematic drawing of stage 3–9 archegonia development. Inner cells are shaded in yellow. The pre‐egg/egg is marked with an asterisk. (b) *PpTARA::GFPGUS‐1/2* reporter output during WT archegonia development. For each stage, a merged image of confocal channels detecting green fluorescent protein (green) and chloroplast autofluorescence (magenta) is shown. (c) Auxin sensing visualized by PpR2D2‐2 output in different stages of archegonia from WT and *Pptara* (*PpR2D2‐2 Pptara‐1*) backgrounds. For each organ, a maximum‐intensity projection of a Z‐stack with confocal channels detecting mDII‐nVENUS (green) and DII‐nTdTOMATO (magenta) merged is shown. Microscopy settings and image processing are identical between the different organs. A high green : magenta signal ratio indicates high auxin sensing (for details, see Thelander *et al*., 2019). For related signal quantification data, see Supporting Information Figs S16 and S17. (d) Differential interference contrast images of representative stage 6–9 archegonia from WT, *Pptara‐1/2*, *Pptaratarb‐1/2* and *Pptaratarc‐1/2* to demonstrate mutant phenotypes. Inner cells and their borders have been shaded in yellow for clarity. Separate boxes show a different focal plane of the boxed area in the neighboring organ. See also Fig. S18.

Although the first morphological sign of female archegonia emergence only become evident around 10 d post‐induction (dpi) at a position separated from the developing antheridia bundle by a leaf (Landberg *et al*., 2013), *PpTARC* expression is activated in a single cell initiating a *de novo* female stem cell region as early as around 7 dpi (Fig. 5a,b). This cell, situated where the abaxial face of the last leaf formed by the vegetative shoot apex connects to the shoot stem, then undergoes two radial anticlinal divisions to form a broad three‐celled ridge able to produce both archegonia and leaves (Fig. 5a,b). The fact that occasional lateral vegetative shoot branches also emerge from leaf axils suggests that cells in this position may be predisposed for stem cell respecification (Coudert *et al*., 2015). While a first leaf initial appears to emerge from one of the flanking cells in the triplet, the first archegonium is produced by a distal division of the middle cell in the triplet, which show *PpTAR* activity (Fig. 5a,b). In the actual organ initial *PpTARA* expression has become more dominant compared to *PpTARC* (Figs 5b, 6b). Despite the *PpTAR* expression the stem cells show extremely low auxin sensing (Fig. 5c) and all *Pptar* mutants produce archegonia initials at the same positions and timing as WT (data not shown). This suggests that auxin is dispensable for the process, unless additional biosynthesis genes, or pathways, are involved. Their *de novo* formation from leaf axils may suggest that archegonia bundles represent lateral shoots which instantly enter a female reproductive program.

### Auxin sensing dependent on *PpTAR*‐mediated synthesis controls cell division activity to ensure proper patterning and radial expansion of archegonia

A mature archegonium consists of an ovoid body with a cavity harboring the egg and a slender neck with a central canal connecting the cavity to the outside to allow sperm entrance (Landberg *et al*., 2013; Fig. 6a). Stage 2 archegonia development is essentially identical to that of antheridia and is driven by the cell division activity of a two‐faced apical stem cell (Fig. 5a). At this stage *PpTARA*, and to some extent *PpTARC*, is expressed throughout the WT organ and auxin sensing is successively increased towards the organ apex in a *PpTAR*‐dependent manner (Figs 5b, 6b,c, S16), largely mirroring the situation in stage 2 antheridia.

Once inner cells form during stages 3–7 they are organized in a uniseriate file typically consisting of six cells where the basal‐most cell is destined to become the egg (Fig. 6a). In WT, inner cells appear to originate from two sequential inward periclinal divisions of the third cell from the organ tip. The first division takes place at stage 3 to produce an inner cell which undergoes transverse anticlinal divisions to give rise to the egg, the upper basal cell and the two longer basal canal cells (Fig. 6a). The second division takes place in stage 5 and produces a short upper canal cell which may duplicate through a transverse anticlinal division (Fig. 6a). The outer cells in the upper part of the *Pptaratarb* archegonia neck frequently undergo ectopic inwards periclinal divisions from stage 5, suggesting that auxin normally restricts neck‐cell proliferation. Some ectopic cells retain a neck cell identity while others take on a canal cell identify resulting in two or more canal cells positioned alongside each other, often misaligned and with a seemingly stochastic variation in length (Fig. 6d). Ectopic canal cells are also seen in *Pptaratarc* archegonia but here localized mainly to the basal part of the neck or even adjacent to the egg cell cavity (Fig. 6d). None of these defects could be detected in any single mutant.

At stages 7 and 8, cell division has completely ceased in WT while it is still evident in mutant archegonia tips. In *Pptara*, a subapical cell now typically undergoes a third periclinal division producing an ectopic apical canal cell while other outer tip cells undergo ectopic radial anticlinal divisions contributing to abnormal organ tip expansion (Fig. 6d). Because *PpTARA* is expressed in the tips of stage 7 and 8 archegonia where it generates high auxin sensing, we conclude that *PpTAR*‐mediated auxin sensing is important to terminate tip‐cell division at these stages (Fig. 6b,c; Fig. S17). These defects are enhanced in the two double mutants. At stage 8, before organ opening at the tip, all inner cells except the egg degrade to clear the central neck canal in WT. Degradation of the abnormal *Pptaratarb* canal cell population typically results in a markedly wide apical cavity instead of the evenly sized canal seen in the WT (Fig. 6d). Most mutant organ tips open in stage 9 even if openings are typically wider and lined by more cells than in the WT (Fig. 6d).

### Auxin sensing dependent on PpTAR‐mediated synthesis promotes elongation of archegonia neck cells

The length of the archegonium neck is significantly reduced in *Pptara*, *Pptaratarb* and *Pptaratarc* (Figs 6d, S18a). As the number of outer neck cells along their apical–basal axis is unaffected, the phenotype is due to cell elongation defects (Figs 6d, S18b). This fits well with the weak neck cell signals from the *PpTARA* reporter and the fact that auxin sensing, largely dependent on *PpTARA*, peaks in basal neck cells at stages 6–7 when cell elongation takes place (Figs 6b,c, S17). The defect becomes enhanced in *Pptaratarb* but not in *Pptaratarc*, suggesting that only *PpTARA* and *PpTARB* contribute to neck cell elongation (Figs 6c,d, S18).

### Egg maturation is characterized by low auxin activity

At stage 5, the pre‐egg starts to enlarge before it undergoes an asymmetric transverse anticlinal division to produce a large basal egg and small upper basal cell (Fig. 6a). At stage 8, when the upper basal cell and the canal cells above it degrade, the egg loses its cell wall and physical connections to cells surrounding it (Fig. 6a). Auxin sensing in the pre‐egg is relatively high and *PpTARA*‐dependent at stage 4/5, but becomes reduced to low levels even before the asymmetric division giving rise to the egg cell (Figs 6c, S17). This level is not further decreased in the *Pptara* mutant (Figs 6c, S17). Auxin sensing remains extremely low in the egg cell until maturity, suggesting that, in line with male gametes, the female gamete maturation and function is linked to low auxin activity (Figs 6c, S17).

The *Pptara* mutant produces normal looking egg cells and the female fertility rate is still considerable (Fig. 6d; Table 2), which fits with the observation that the low auxin sensing in the WT pre‐egg and egg is not further reduced when *PpTARA* is deleted (Figs 6c, S17). By contrast, the double mutants both suffer severe egg cell defects and show no (*Pptaratarb*) or very low (*Pptaratarc*) female fertility and the few sporophytes formed are malformed or blocked in development (Fig. 6d; Table 2). An obvious pre‐egg (e.g. rounded and enlarged) possible to discriminate from canal cells at stages 5–6 is missing from the majority of double mutant archegonia, which frequently results in empty cavities or cavities containing an abnormal and severely condensed egg cell at stage 9 (Fig. 6d). Although we cannot exclude effects on the differentiation or function of egg cells as such, it appears likely that the frequent lack of egg cells, but not necessarily the egg abnormalities, are indirect consequences of the disturbed pattering caused by ectopic cell division activity already discussed.

**Table 2:** Outcome of genetic crosses involving various *Physcomitrium (Physcomitrella) patens Pptar* mutants and WT.

Female parent egg donor	Male parent sperm donor	Shoots analyzed	Frequency of shoots initiating sporophyte development (%)	Frequency of immature or malformed sporophytes among those initiated (%)
WT	WT	194	99	8
*Pptara‐1*	*Pptara‐1*	128	40	14
*Pptara‐1*	WT	98	53	2
*Pptaratarb‐1*	*Pptaratarb‐1*	133	0	–
*Pptaratarb‐1*	WT	165	0	–
*Pptaratarb‐2*	*Pptaratarb‐2*	513	0	–
*Pptaratarb‐2*	WT	399	0	–
*Pptaratarc‐1*	*Pptaratarc‐1*	329	5	100
*Pptaratarc‐1*	WT	425	15	98
*Pptaratarc‐2*	*Pptaratarc‐2*	322	5	100
*Pptaratarc‐2*	WT	138	13	100

## Discussion

We show that *PpTAR* and *PpYUC* homologs encode functional auxin biosynthesis enzymes and constitute a major route for auxin biosynthesis in moss. Together with similar findings from flowering plants and a liverworth, two other deep branches of land plants, this confirms that the IPyA pathway dates back at least to the common ancestors of all land plants (Ljung, 2013; Eklund *et al*., 2015; Romani, 2017). The pathway is of major importance for developmental regulation in moss, and restriction of *PpTAR* activity results in decreased auxin sensing linked to severe developmental abnormalities. Preliminary observations of selected *PpYUC* reporters and mutants fit well with the more complete *PpTAR* data presented in this study (Fig. S19). While this study focused on reproductive development, the IPyA pathway probably plays important roles throughout the life cycle. Our failure to produce a triple mutant in which the three major *PpTAR* genes (A–C) are deleted probably reflects an essential function.

The acquirement of nuclear auxin sensing may have provided ancestral plants with a means for intercellular coordination of cell proliferation and differentiation to facilitate focal growth (Flores‐Sandoval *et al*., 2018). Our data suggest an underlying mechanism for how this works. Proliferation of a moss apical stem cell requires low auxin sensing but at the same time this cell synthesizes auxin to control different aspects of differentiation of its progeny.

The formation of gametangia and leaves at the moss shoot apex is initially driven by two‐faced apical stem cells cleaving off cells backwards in two parallel files. In these cells, auxin sensing is initially extremely low, while continuous *PpTAR*‐dependent auxin synthesis contributes to a successive build‐up of sensing as organs grow larger (this study; Thelander *et al*., 2019; Fig. S20). Once sensing has reached a certain threshold, it terminates apical stem cell division, indicating that a successive build‐up of sensing by local auxin biosynthesis could represent a simple general mechanism to control the window during which two‐faced apical stem cells remain active. After inactivation of the two‐faced stem cells, organ development is driven by a mix of divisions, expansions and fate changes affecting the apical stem cell derivatives (Figs 4a, 6a; Harrison *et al*., 2009). *PpTAR*‐dependent auxin sensing also regulates these processes by restricting periclinal and radial anticlinal divisions while promoting apical–basal cell elongation.

Unlike gametangia and leaf apical stem cells, the three‐faced apical stem cell of the shoot retains *PpTAR* expression combined with extremely low auxin sensing indeterminately, probably blocking stem cell arrest. As PpPIN auxin exporters have been hypothesized to remove auxin from the meristem (Bennett *et al*., 2014), although *PpPIN* reporters fail to detect their precise localization (data not shown; Viaene *et al*., 2014), the inability of the stem cell to sense auxin could be caused by auxin drainage in combination with active repression of auxin responses. In *M. polymorpha*, a repressive type B ARF is active in the apical region of the thallus, which also produce auxin via the IPyA pathway to control, for example, the dormancy of gemmae produced by its progeny (Eklund *et al*., 2015; Kato *et al*., 2020). Local IPyA‐mediated auxin biosynthesis may thus represent an ancestral general mechanism for apical stem cells to control where in relation to their own position critical differentiation processes affecting their progeny occur. Where auxin produced by the moss shoot apex is sensed is unclear. However, *Pptaratarc* shoots, lacking the two *PpTAR* genes significantly expressed in vegetative shoot apices (Fig. 2b), are dwarfed and the frequency of lateral branching is increased, revealing that auxin promotes cell expansion and suppresses respecification of epidermal cells into competing lateral shoot apical stem cells (Fig. S11; data not shown; Fujita *et al*., 2008; Coudert *et al*., 2015). Some support for both functions is found in reproductive organs as *PpTAR*‐dependent auxin sensing is important to suppress ectopic formation of antheridium apical stem cells and to promote apical–basal cell expansion in the archegonia neck.

Auxin sensing drops dramatically during gamete differentiation, explaining why both eggs and sperm are characterized by strikingly low auxin activity. This coincides with declining *PpTAR* expression and activation of auxin transport. Spermatogenous cells express *PpPIND*, a short endoplasmic reticulum‐localized transporter, limiting the pool of available auxin in the nucleus (Fig. S21; Viaene *et al*., 2014). The pre‐egg and egg instead express the long auxin exporters PpPINA, B and C from stage 6, suggesting that they are actively drained of auxin (Fig. S22; Landberg *et al*., 2013; Viaene *et al*., 2014). As *PpTAR* expression remains active in the egg to stage 7, well after *PpPINA‐C* activation and the loss of auxin sensing (stage 5), auxin export may prepare the egg and/or surrounding tissues for fertilization, for example by activating degradation of the egg cell wall (Figs 6b,c, S17). This hypothesis is based on the observation that *PpPINA* expression in antheridia and archegonia tips coincides with organ opening facilitated by cell wall degradation (Landberg *et al*., 2013).

If the strikingly low auxin activity of the moss gametes is a prerequisite for their development, and/or whether it is critical for successful fertilization or even downstream embryo development is not clear. The Arabidopsis egg and central cell are characterized by low auxin activity, which is broken upon double fertilization to drive development of the embryo and endosperm, but also to trigger growth and development of the surrounding mother tissues (Figueiredo *et al*., 2016). The malformed/aborted sporophytes produced by *Pptaratarc* both after selfing and fertilization by WT sperm show that auxin synthesis is important also for moss embryo/sporophyte development, and may be related to problems with the interaction between embryo and the surrounding mother tissues.

## Author contributions

JŠ and KLjung conducted and analyzed the auxin metabolite measurements and revised the manuscript. KLandberg and MT conducted all other experiments and analyzed the data. KLandberg, ES and MT designed the experiments, interpreted the results and wrote the manuscript.

## Supporting information


**Fig. S1** *PpTARA*, *PpTARF*, *PpYUCA* and *PpYUCC* overexpressor lines in WT background: construct design, PCR verification and expression levels.
**Fig. S2** *PpTARA* overexpressor lines in *Pptara‐1* mutant background: construct design, PCR verification and expression levels.
**Fig. S3** *PpTARA*, *PpTARB*, *PpTARC*, *PpTARD* and *PpYUCF* transcriptional reporter lines in WT background: construct design and PCR verification.
**Fig. S4** *PpTARA‐D* and *PpYUCA‐F* single knockout lines in WT background: principal construct design and PCR verification.
**Fig. S5** PCR verification of *PpTAR* double and triple knockout lines.
**Fig. S6** Confirmation of knockout lines carrying the PpR2D2 reporter.
**Fig. S7** Intron positions in coding regions are conserved in *Arabidopsis* and *P. patens* *TAR* genes of both the TAA clade and the AtTAR3/4 clade.
**Fig. S8** The number of introns in *YUC* gene coding regions differ in both Arabidopsis and *P. patens* but the positions of introns that do exist are conserved.
**Fig. S9** The diameter of *Pptara* protonemal colonies is reduced.
**Fig. S10** Relative expression of *PpTAR* genes in chloronema.
**Fig. S11** *Pptaratarc* shoots are dwarfed but otherwise develop normally.
**Fig. S12** Quantitative PpR2D2 output as a measure of auxin sensing during stage 2 antheridia development.
**Fig. S13** Complementation of *Pptara* reproductive organ phenotype by *PpTARA* overexpression.
**Fig. S14** Quantitative PpR2D2 output as a measure of auxin sensing during stage 3–9 antheridia development.
**Fig. S15** A subset of inner cells do not proliferate in the *Pptaratarc* double mutant.
**Fig. S16** Quantitative PpR2D2 output as a measure of auxin sensing during stage 2 archegonia development.
**Fig. S17** Quantitative PpR2D2 output as a measure of auxin sensing during stage 3–9 archegonia development.
**Fig. S18** Archegonia neck lengths are reduced in *Pptar* mutants due to a cell elongation defect.
**Fig. S19** *PpYUC* expression patterns and knockout phenotype resemble those of *PpTAR* in reproductive organs.
**Fig. S20** Auxin sensing in the apical stem cell of young vegetative leaves is successively increased in a *PpTAR*‐dependent manner.
**Fig. S21** *PpPIND* is expressed in spermatogenous cells of antheridia.
**Fig. S22** Long *PpPINs* are expressed in the pre‐egg/egg from around stage 5 of archegonia development.
**Methods S1** Generation of overexpression constructs and lines.
**Methods S2** Generation of transcriptional reporter constructs and lines.
**Methods S3** Generation of knockout constructs and lines.
**Methods S4** Generation of knockout lines carrying the PpR2D2 reporter.
**Methods S5** RT‐qPCR to determine *PpTAR* transcript abundance.
**Table S1** Constructs produced and used in the study.
**Table S2** Primers used in study.
**Table S3** Level of auxin metabolites in wildtype *P. patens* and in stated overexpressor and mutant lines.
Please note: Wiley Blackwell are not responsible for the content or functionality of any supporting information supplied by the authors. Any queries (other than missing material) should be directed to the *New Phytologist* Central Ofﬁce.
