# Supplementary material for: Studies of moss reproductive development indicate that auxin biosynthesis in apical stem cells may constitute an ancestral function for focal growth control
Source: New Phytol. 2020 Oct 4;229(2):845–60. doi: 10.1111/nph.16914 (PMC7821132; doi:10.1111/nph.16914)
Supplement: Supplementary file 1 — Fig. S1 PpTARA, PpTARF, PpYUCA and PpYUCC overexpressor lines in WT background: construct design, PCR verification and expression levels. Fig. S2 PpTARA overexpressor lines in Pptara‐1 mutant background: construct design, PCR verification and expression levels. Fig. S3 PpTARA, PpTARB, PpTARC, PpTARD and PpYUCF transcriptional reporter lines in WT background: construct design and PCR verification. Fig. S4 PpTARA‐D and PpYUCA‐F single knockout lines in WT background: principal construct design and PCR verification. Fig. S5 PCR verification of PpTAR double and triple knockout lines. Fig. S6 Confirmation of knockout lines carrying the PpR2D2 reporter. Fig. S7 Intron positions in coding regions are conserved in Arabidopsis and P. patens TAR genes of both the TAA clade and the AtTAR3/4 clade. Fig. S8 The number of introns in YUC gene coding regions differ in both Arabidopsis and P. patens but the positions of introns that do exist are conserved. Fig. S9 The diameter of Pptara protonemal colonies is reduced. Fig. S10 Relative expression of PpTAR genes in chloronema. Fig. S11 Pptaratarc shoots are dwarfed but otherwise develop normally. Fig. S12 Quantitative PpR2D2 output as a measure of auxin sensing during stage 2 antheridia development. Fig. S13 Complementation of Pptara reproductive organ phenotype by PpTARA overexpression. Fig. S14 Quantitative PpR2D2 output as a measure of auxin sensing during stage 3–9 antheridia development. Fig. S15 A subset of inner cells do not proliferate in the Pptaratarc double mutant. Fig. S16 Quantitative PpR2D2 output as a measure of auxin sensing during stage 2 archegonia development. Fig. S17 Quantitative PpR2D2 output as a measure of auxin sensing during stage 3–9 archegonia development. Fig. S18 Archegonia neck lengths are reduced in Pptar mutants due to a cell elongation defect. Fig. S19 PpYUC expression patterns and knockout phenotype resemble those of PpTAR in reproductive organs. Fig. S20 Auxin sensing in the apical stem cell of you [file NPH-229-845-s001.pdf]

***New Phytologist* Supporting Information**

Article title: Studies of moss reproductive development indicate that auxin biosynthesis in apical stem cells may constitute an ancestral function for focal growth control

Authors: Landberg Katarina, Šimura Jan, Ljung Karin, Sundberg Eva and Thelander Mattias

Article acceptance date: 23 August 2020

The following Supporting Information is available for this article:

Method S1 Generation of overexpression constructs and lines

Method S2 Generation of transcriptional reporter constructs and lines

Method S3 Generation of knockout constructs and lines

Method S4 Generation of knockout lines carrying the PpR2D2 reporter

Method S5 RT-qPCR to determine *PpTAR* transcript abundance

Figure S1 *PpTARA*, *PpTARF*, *PpYUCA* and *PpYUCC* overexpressor lines in WT background: Construct design, PCR verification and expression levels.

Figure S2 *PpTARA* overexpressor lines in *Pptara-1* mutant background: Construct design, PCR verification and expression levels.

Figure S3 *PpTARA*, *PpTARB*, *PpTARC*, *PpTARD* and *PpYUCF* transcriptional reporter lines in WT background: Construct design and PCR verification.

Figure S4 *PpTARA-D* and *PpYUCA-F* single knockout lines in WT background: Principal construct design and PCR verification.

Figure S5 PCR verification of *PpTAR* double and triple knockout lines.

Figure S6 Confirmation of knockout lines carrying the PpR2D2 reporter.

Figure S7 Intron positions in coding regions are conserved in *Arabidopsis* and *P. patens TAR* genes of both the TAA clade and the AtTAR3/4 clade.

Figure S8 The number of introns in *YUC* gene coding regions differ in both Arabidopsis and *P. patens* but the positions of introns that do exist are conserved.

Figure S9 The diameter of *Pptara* protonemal colonies is reduced.

Figure S10 Relative expression of *PpTAR* genes in chloronema

Figure S11 *Pptaratarc* shoots are dwarfed but otherwise develop normally

Figure S12 Quantitative PpR2D2 output as a measure of auxin sensing during stage 2 antheridia development.

Figure S13 Complementation of *Pptara* reproductive organ phenotype by *PpTARA* OE

Figure S14 Quantitative PpR2D2 output as a measure of auxin sensing during stage 3 to 9 antheridia development.

Figure S15 A sub-set of inner cells do not proliferate in the *Pptaratarc* double mutant.

Figure S16 Quantitative PpR2D2 output as a measure of auxin sensing during stage 2 archegonia development.

Figure S17 Quantitative PpR2D2 output as a measure of auxin sensing during stage 3 to 9 archegonia development.

Figure S18 Archegonia neck lengths are reduced in *Pptar* mutants due to a cell elongation defect.

Figure S19 *PpYUC* expression patterns and knockout phenotype resemble those of *PpTAR* in reproductive organs**.**

Figure S20 Auxin sensing in the apical stem cell of young vegetative leaves is successively increased in a *PpTAR*-dependent manner.

Figure S21 *PpPIND* is expressed in spermatogenous cells of antheridia.

Figure S22 Long *PpPINs* are expressed in the pre-egg / egg from around stage 5 of archegonia development.

Table S1 Constructs produced and used in the study

Table S2 Primers used in study

Table S3 Level of auxin metabolites in wildtype *P. patens* and in stated over-expressor (OE) and mutant lines

Supplementary references

Method S1. Generation of overexpression (OE) constructs and lines. To produce the vector pMT258, a 1429 bp Rice actin promoter fragment amplified with primers SS596/598 was trimmed with *Hind*III/*Sal*I and cloned between the same sites of plasmid pUK-Pp108+Hsp-GW+npt (gift from Michael Prigge and Mark Estelle). The resulting vector pMT258 carries a G418 selection cassette (G418R) and allows coding sequences to be cloned behind the Rice actin promoter for subsequent integration into the *Pp108* locus. To produce the *PpTARF* OE construct pMT261, a 1488 bp *PpTARF* CDS fragment was amplified from cDNA with primers SS378/SS379, trimmed with *Avr*II/*Sal*I, and cloned between the same sites of pMT258. To produce the *PpYUCC* OE construct pMT262, a 1317 bp *PpYUCC* CDS fragment was amplified from cDNA with primers SS372/373, trimmed with *Avr*II/*Sal*I, and cloned between the same sites of pMT258. To produce the *PpYUCA* OE construct pMT266, a 1323 bp *PpYUCA* CDS fragment was amplified from cDNA with primers SS370/371, trimmed with *Avr*II/*Sal*I, and cloned between the same sites of pMT258. To produce the OE vector pMT272 carrying a zeocin selection cassette (ZeoR), G418R in pMT258 was excised by *Avr*II/*Sal*I and exchanged for ZeoR excised from plasmid 35S-Zeo by *Spe*I/*Xho*I. To produce the *PpTARA* OE construct pMT281, a 1554 bp *PpTARA* CDS fragment was amplified from cDNA with primers SS469/470, trimmed with *Xma*JI, and cloned into the same site of pMT272. All OE constructs were linearized with *Sfi*I before transformation into WT. The *PpTARA* OE construct was also transformed into the *Pptara-1* mutant background. Correct integration was confirmed by PCR and level of overexpression was determined by qPCR (Fig. S1, S2). Resulting constructs and sequences of primers used for cloning and genotyping are summarized in Tables S1 and S2, respectively.

Method S2. Generation of transcriptional reporter constructs and lines. To construct promoter-based reporters, the vector pMT211 carrying a hygromycin selection cassette (HygR) and allowing promoters to be cloned ahead of a *GFP-GUS* reporter gene for subsequent integration into the *Pp108* locus was used (Thelander et al., 2019). To produce the *PpTARA* reporter construct pMT243, a 1808 bp *PpTARA* promoter fragment was amplified from gDNA with primers SS220/SS221, trimmed with *Bam*HI/*Nco*I, and cloned between the same sites of pMT211. To produce the *PpTARB* reporter construct pMT241, a 2789 bp *PpTARB* promoter fragment was amplified from gDNA with primers SS222/223, trimmed with *Bsp*HI, and cloned into the same site of pMT211. To produce the *PpTARC* reporter construct pMT254, a 2987 bp *PpTARC* promoter fragment was amplified from gDNA with primers SS224/225, trimmed with *Bam*HI/*Nco*I, and cloned between the same sites of pMT211. To produce the *PpTARD* reporter construct pMT237, a 2446 bp *PpTARD* promoter fragment was amplified from gDNA with primers SS226/227, trimmed with *Hind*III/*Nco*I, and cloned between the same sites of pMT211. To produce the *PpYUCF* reporter construct pMT246, a 2120 bp *PpYUCF* promoter fragment was amplified from gDNA with primers SS244/245, trimmed with *Bam*HI/*Nco*I, and cloned between the same sites of pMT211. All reporter constructs were linearized with *Sfi*I before transformation into WT. Correct integration was confirmed by PCR (Fig. S3). Resulting constructs and sequences of primers used for cloning and genotyping are summarized in Tables S1 and S2, respectively.

Method S3. Generation of knockout (KO) constructs and lines. All KO constructs were designed to delete the entire coding region of the gene of interest and genomic DNA was used as the template in all PCRs to amplify 5' and 3' flanking regions. Most KO constructs were produced by Gateway facilitated 3-fragment recombination (www.thermofisher.com). In each

such case, LR recombination was used to link a 5’ flanking fragment (cloned in entry vector pDONR P1-P4), a selection cassette (cloned in entry vector pDONR P4r-P3r), and a 3’ flanking fragment (cloned in entry vector pDONR P3-P2 by) in the destination vector pDEST14 (www.thermofisher.com). Three different selection cassette entry clones were produced and used: Primers SS265/266 were used to amplify and clone HygR from plasmid pMT123 (Thelander et al., 2004) to produce entry clone pMT222, primers SS611/612 were used to amplify and clone G418R from plasmid pMT164 (Thelander et al., 2007) to produce entry clone pDONR4r-3r-G418, and primers SS613/SS614 were used to amplify ZeoR from plasmid 35S-Zeo to produce entry clone pDONR4r-3r-Zeo. To produce the *PpTARC* KO construct pMT229 linearized with *Xho*I prior to transformation, a 1024 bp *PpTARC* 5’ fragment amplified with primers SS269/270 was linked to G418R and a 1007 bp *PpTARC* 3’ fragment amplified with primers SS271/SS272. To produce the *PpYUCA* KO construct pMT263 linearized with *Sal*I prior to transformation, a 1136 bp *PpYUCA* 5’ fragment amplified with primers SS325/326 was linked to ZeoR and a 906 bp *PpYUCA* 3’ fragment amplified with primers SS368/369. To produce the *PpYUCB* KO construct pMT276 linearized with *Sal*I prior to transformation, a 1081 bp *PpYUCB* 5’ fragment amplified with primers SS424/425 was linked to ZeoR and a 1073 bp *PpYUCB* 3’ fragment amplified with primers SS426/SS476. To produce the *PpYUCC* KO construct pMT232 linearized with *Xho*I prior to transformation, a 1055 bp *PpYUCC* 5’ fragment amplified with primers SS281/282 was linked to G418R and a 1095 bp *PpYUCC* 3’ fragment amplified with primers SS283/284. To produce the *PpYUCD* KO construct pMT236 linearized with *Xho*I prior to transformation, a 1009 bp *PpYUCD* 5’ fragment amplified with primers SS285/286 was linked to ZeoR and a 960 bp *PpYUCD* 3’ fragment amplified with primers SS287/288. To produce the *PpYUCE* KO construct pMT233 linearized with *Sal*I prior to transformation, a 938 bp *PpYUCE* 5’ fragment amplified with primers SS290/292 was linked to G418R and a 984 bp *PpYUCE* 3’ fragment amplified with primers SS293/294. To produce the *PpYUCF* KO construct pMT235 linearized with *Sal*I prior to transformation, a 1007 bp *PpYUCF* 5’ fragment amplified with primers SS295/296 was linked to HygR and a 815 bp *PpYUCF* 3’ fragment amplified with primers SS298/300.

The *PpTARA*, *PpTARB* and *PpTARD* KO constructs were produced by traditional consecutive ligation steps. To produce the *PpTARA* HygR KO construct pMT210 linearized with *Sna*BI/*Sac*I prior to transformation, a *PpTARA* 5’ flanking fragment amplified with primers SS603/604 was trimmed to 1086 bp with *Xho*I/*Hind*III and ligated between the same sites of plasmid pMT123 where after a *PpTARA* 3’ flanking fragment amplified with primers SS605/606 was trimmed to 952 bp with *Xba*I/*Sac*I and ligated between the same sites of the product of the first ligation. To produce the *PpTARB* G418R KO construct pEP54 linearized with *Bam*HI/*Pac*I prior to transformation, a *PpTARB* 5’ flanking fragment amplified with primers SS607/608 was trimmed to 1030 bp with *Bam*HI/*Xho*I and ligated between the same sites of plasmid pMT164 where after a *PpTARB* 3’ flanking fragment amplified with primers SS609/610 was trimmed to 894 bp with *Spe*I/*Pac*I and ligated between the same sites of the product of the first ligation. To produce the *PpTARD* HygR KO construct pMT207 linearized with *Xho*I/*Sac*I prior to transformation, a *PpTARD* 5’ flanking fragment amplified with primers SS599/600 was trimmed to 907 bp with *Xho*I/*Hind*III and ligated between the same sites of plasmid pMT123 where after a *PpTARD* 3’ flanking fragment amplified with primers SS601/602 was trimmed to 1011 bp with *Spe*I/*Sac*I and ligated between the same sites of the product of the first ligation.

All KO constructs were transformed into WT to produce a full set of *PpTARA-D* and *PpYUCA-F* single KO lines. The *PpTARB* KO construct pEP54 was also transformed into the confirmed *Pptara-1* mutant to produce *Pptaratarb* double KO lines. Other *PpTAR* double and triple mutants (*Pptaratarc*, *Pptarctard* and *Pptarbtarctard*) were produced by crosses from confirmed single mutant lines as described in Thelander et al., 2019. Lines resulting from new transformations were checked for correct construct integration by PCRs confirming the 5' junction, the 3' junction and the loss of internal gene sequences while the genotypes of lines resulting from crosses were confirmed only by a PCR demonstrating the loss of internal gene sequences (Fig. S4, S5). Constructs and sequences of primers used for cloning and genotyping are summarized in Tables S1 and S2, respectively.

Method S4. Generation of knockout lines carrying the PpR2D2 reporter. PpR2D2-2 and PpR2D2-3 (Thelander et al., 2019) were crossed to *Pptara-1* and *Pptaratarc-1* where after colonies from regenerating spores were screened for loss of *PpTARC*, resistance to selection agents, *Pptar* mutant phenotoypes, and PpR2D2 signals to identify mutant offspring carrying the PpR2D2 reporter construct (Fig. S6). Sequences of primers used for genotyping are summarized in Table S2.

Method S5. RT-qPCR to determine *PpTAR* transcript abundance. To determine *PpTARA-D* transcript abundance in various WT tissues material was harvested in triplicates, snap frozen and stored in -80°C awaiting RNA isolation. Chloronema-enriched protonema was harvested from cellophane-overlaid BCD media grown under standard conditions for 5 days since the last subcultivation by blending. To collect shoot apices and reproductive organs, colonies were grown on thick BCD plates in standard conditions for 45 days before transfer to inductive conditions. Shoot apices including only the youngest leaves were harvested before or 2, 10, 13 or 16 days after transfer to inductive conditions. Antheridia and archegonia bundles were separately harvested after 15-16 and 20 days in inductive conditions, respectively. At these time points, the most mature organs were typically at stage 6-7 and stage 9 (Landberg et al., 2013), respectively, but the bundles also contained a mix of younger organs. Total RNA was extracted from samples using the Picopure RNA extraction kit from Arcturus (Thermo Fisher Scientific). The manufacturer's protocol was followed with two additions: Tissue was homogenized with glass beads in a fast-prep machine for 60 s at full speed and an on-column DNase treatment step (Qiagen, 79254) was included. From each sample, 50 ng of total RNA was converted into cDNA and amplified using the Ovation PicoSL WTA System V2 from NuGen in accordance with instructions from the manufacturer. Quantitative real-time PCRs were based on Maxima SYBR Green/ROX qPCR master mix (Thermo Fisher Scientific, K0221) and 4 ng of amplified cDNA was consistently used in total reaction volumes of 25 µl. Reactions were run on an iQ5 Real-Time PCR system (Bio-Rad, Sundbyberg, Sweden) at 95°C for 10 min followed by 40 cycles of 95°C for 15 sec, 60° for 30 s and 72° for 30 s. To rule out amplification from non-specific targets and contaminating genomic DNA, *PpTARA-D* primers were designed to anneal to regions with limited sequence similarity and one primer in each pair also has an annealing site interrupted by an intron. For normalization, the three reference genes AdePRT, E2 and St-P2a were used. These three reference genes were selected among six candidates described in Le Bail et al., 2013 since they showed the most stable expression in antheridia and archegonia in our hands (data not shown). The primers used were: *PpTARA*, SS501/SS502; *PpTARB*, SS503/SS504; *PpTARC*, SS517/SS518, *PpTARD*, SS519/SS520; *AdePRT*, SS549/SS460; *E2*, SS455/SS456; *ST-P2a*,

SS463/SS464 (for sequences, see Table S2). Melt curve analysis, gel analysis and standard curve analysis confirmed that all primer pairs amplified a single product of the expected size with efficiencies of 100 ± 5 % (data not shown). Fig. 2a,b and Fig. S10a show relative expression calculated with the $2^{-\Delta\Delta CT}$ method using the gene with the highest transcript abundance across the samples as the calibrator. Each data point is based on biological triplicates and error bars represent standard deviation.

To determine transcript abundance in *PpTARA, PpTARF, PpYUCA* and *PpYUCC* OE lines the RNeasy plant mini kit (Qiagen, 74903) was used to prepare total RNA from young protonemal tissue grown under standard conditions in duplicates or triplicates. On column DNAse treatment was performed for all samples (Qiagen, 79254). 1 µg RNA was used for cDNA synthesis using the Superscript III reverse transcript kit (Thermo Fisher Scientific, 18080051). Quantitative real-time PCRs were performed as described above, but with 1 µl cDNA as template per 25 µl reaction. For normalization, *AdePRT* (see above; for *PpTARA*) or actin (Prigge et al., 2010; for *PpTARF*, *PpYUCA* and *PpYUCC*) were used as reference genes. The primers used were: *PpTARA*, SS501/SS502; *PpTARF*, SS434/SS435; *PpYUCA*, SS430/SS431; *PpYUCC*, SS428/SS429; *ACT*, SS29/SS30; *AdePRT*, SS459/460 (for sequences, see Table S2). Fig. S1f-i and Fig. 2c show relative expression of 2-3 biological replicates for each line with the WT, or the OE line showing the lowest expression in cases when transcript were under detection level in WT, set to 1.

Fig. S1. *PpTARA*, *PpTARF*, *PpYUCA* and *PpYUCC* overexpressor lines in WT background: Construct design, PCR verification and expression levels. (**A**) Schematic view of overexpression constructs and the *Pp108* locus to which they were targeted. Arrows mark the approximate annealing sites of primers used for PCR verification in B-E. (**B-E**) PCR verification of 5’ and 3’ junctions to confirm correct integration in lines transformed with the *PpTARA* (B), *PpTARF* (C), *PpYUCA* (D) and *PpYUCC* (E) overexpressor constructs. Expected product sizes are indicated within parenthesis and the sequences of listed primers are shown in Table S2. (**F-I**) Relative *PpTARA* (F), *PpTARF* (G), *PpYUCA* (H) and *PpYUCC* (I) expression levels in selected overexpression lines as revealed by qPCR’s using cDNA from young protonemal tissue as the template. For each line/gene combination the results from three (*PpTARA*) or two (*PpTARF*, *PpYUCA*, *PpYUC*) biological replicates are displayed as bars while the mean of replicates are indicated by a horizontal line and a number. In F (*PpTARA*) and I (*PpYUCC*) the mean WT expression is set to 1. For G and H the overexpressor line with the lowest expression is set to 1 since no expression could be detected for *PpTARF* and *PpYUCA* in the WT protonemal tissue sampled. For primers sequences, see Table S2.

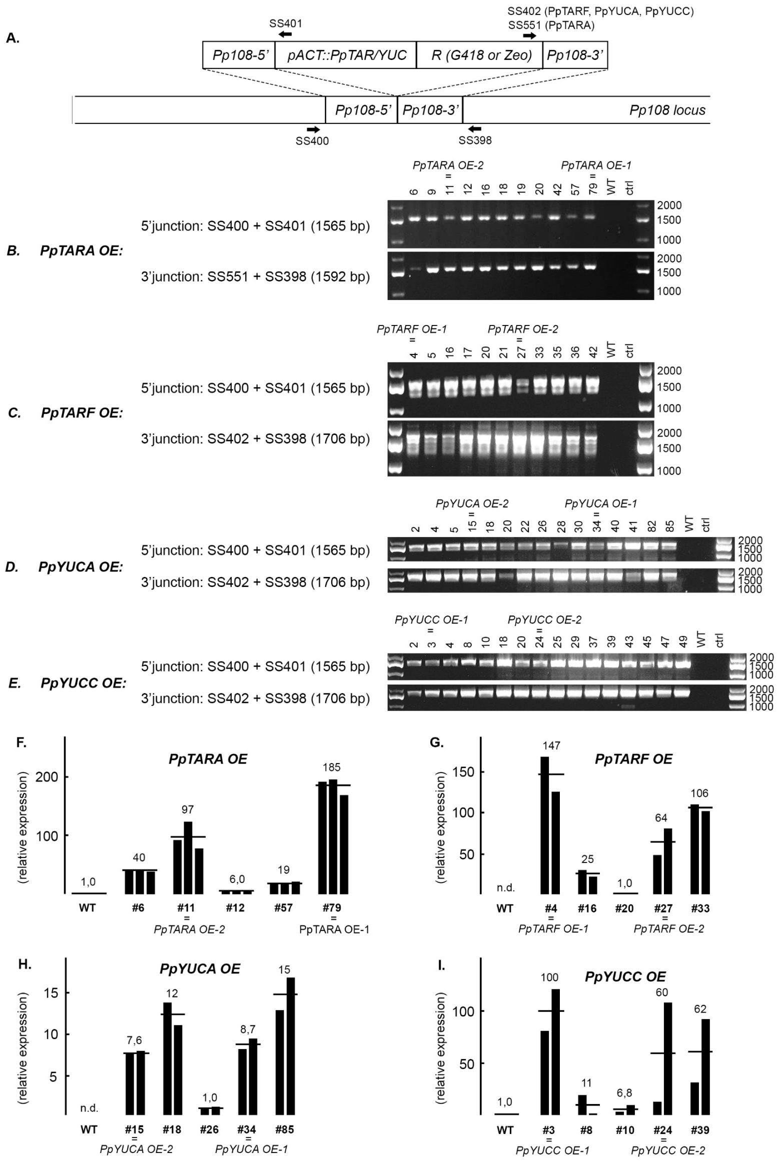

Fig. S2. *PpTARA* overexpressor lines in *Pptara-1* mutant background: Construct design, PCR verification and expression levels. (**A**) Schematic view of overexpression construct and the *Pp108* locus to which it was targeted. Arrows mark the approximate annealing sites of primers used for PCR verification in B. (**B**) PCR verification of 5' and 3' junctions to confirm correct integration. Expected product sizes are indicated within parenthesis and the sequences of listed primers are shown in Table S2. (**C**) Relative *PpTARA* expression levels in selected overexpression lines as revealed by qPCR's using cDNA from young protonemal tissue as the template. For each line the results from three biological replicates are displayed as bars while the mean of replicates are indicated by a horizontal line and a number. The mean WT expression is set to 1. For primers sequences, see Table S2. For primers sequences, see Table S2.

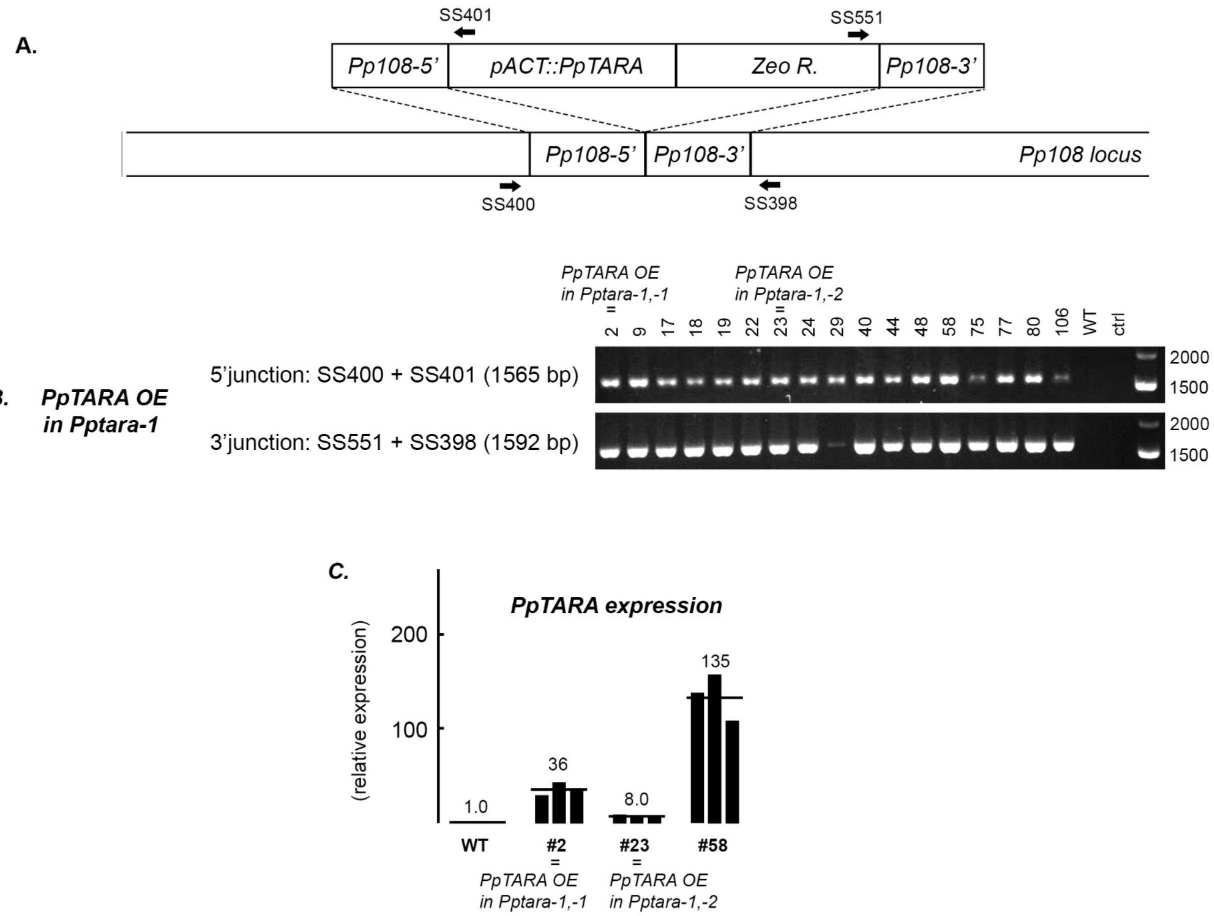

Fig. S3. *PpTARA*, *PpTARB*, *PpTARC*, *PpTARD* and *PpYUCF* transcriptional reporter lines in WT background: Construct design and PCR verification. (**A**) Schematic view of constructs and the *Pp108* locus to which they were targeted. Arrows mark the approximate annealing sites of primers used for PCR verification in B-F. (**B-F**) PCR verification of 5' and 3' junctions to confirm correct integration. Expected product sizes are indicated within parenthesis. For primers sequences, see Table S2.

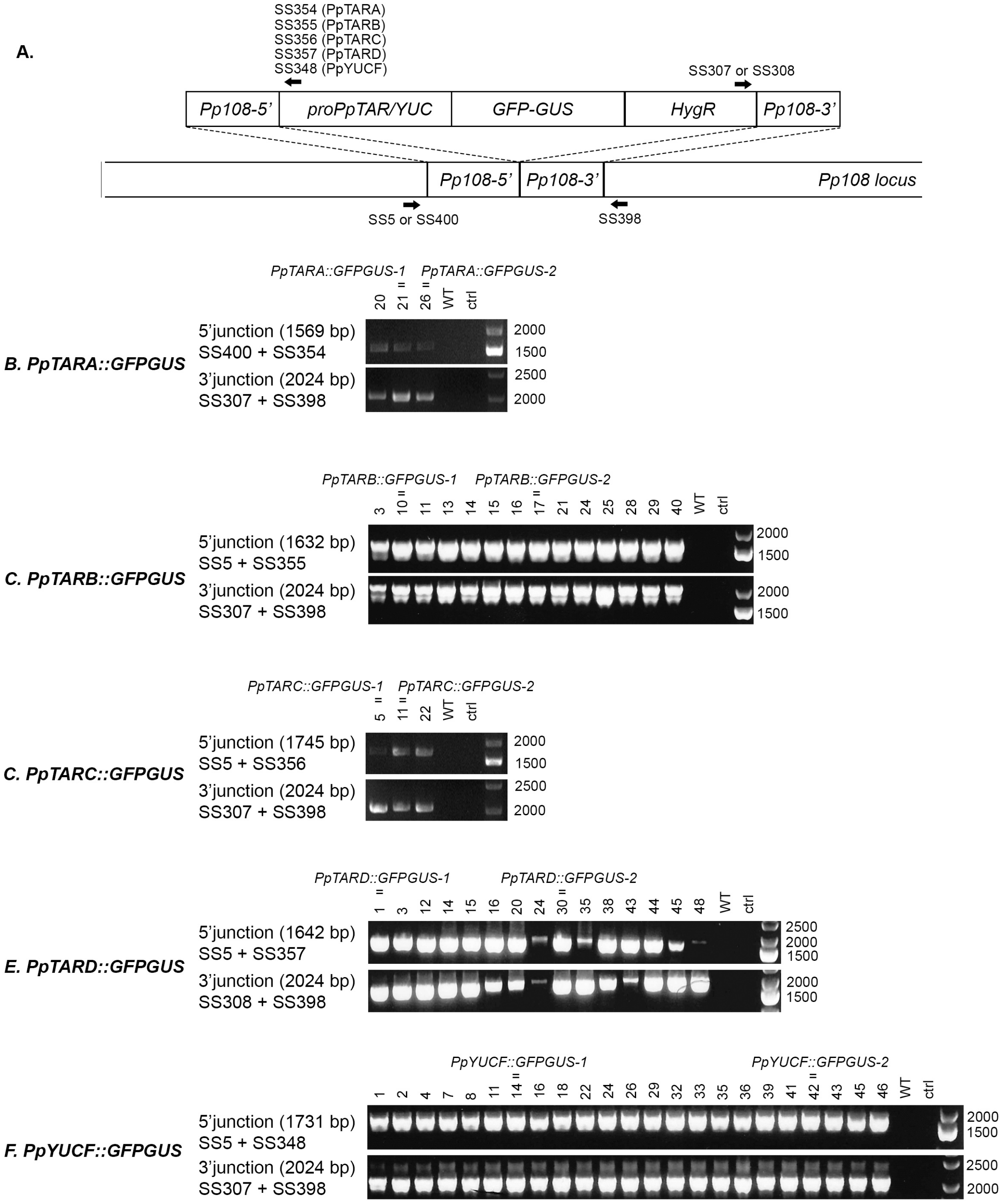

Fig. S4. *PpTARA-D* and *PpYUCA-F* single knockout lines in WT background: Principal construct design and PCR verification. (**A**) Schematic consensus view of constructs and loci of genes of interest (GOI) to which they were targeted. Arrows mark the approximate annealing sites of primers used for PCR reactions to confirm correct integration in B-K. (**B-K**) Results of PCR verification to confirm 5' junctions (5' PCR), 3' junctions (3' PCR) and loss of internal gene sequences (Int. PCR). For each genotype, primer names and expected product sizes are indicated for each of the three PCR types. For primers sequences, see Table S2. Note: Verification of the 3' junction in *PpTARD* KO lines was not possible due to the presence of an extended AT-rich region immediately downstream of the *PpTARD* coding region.

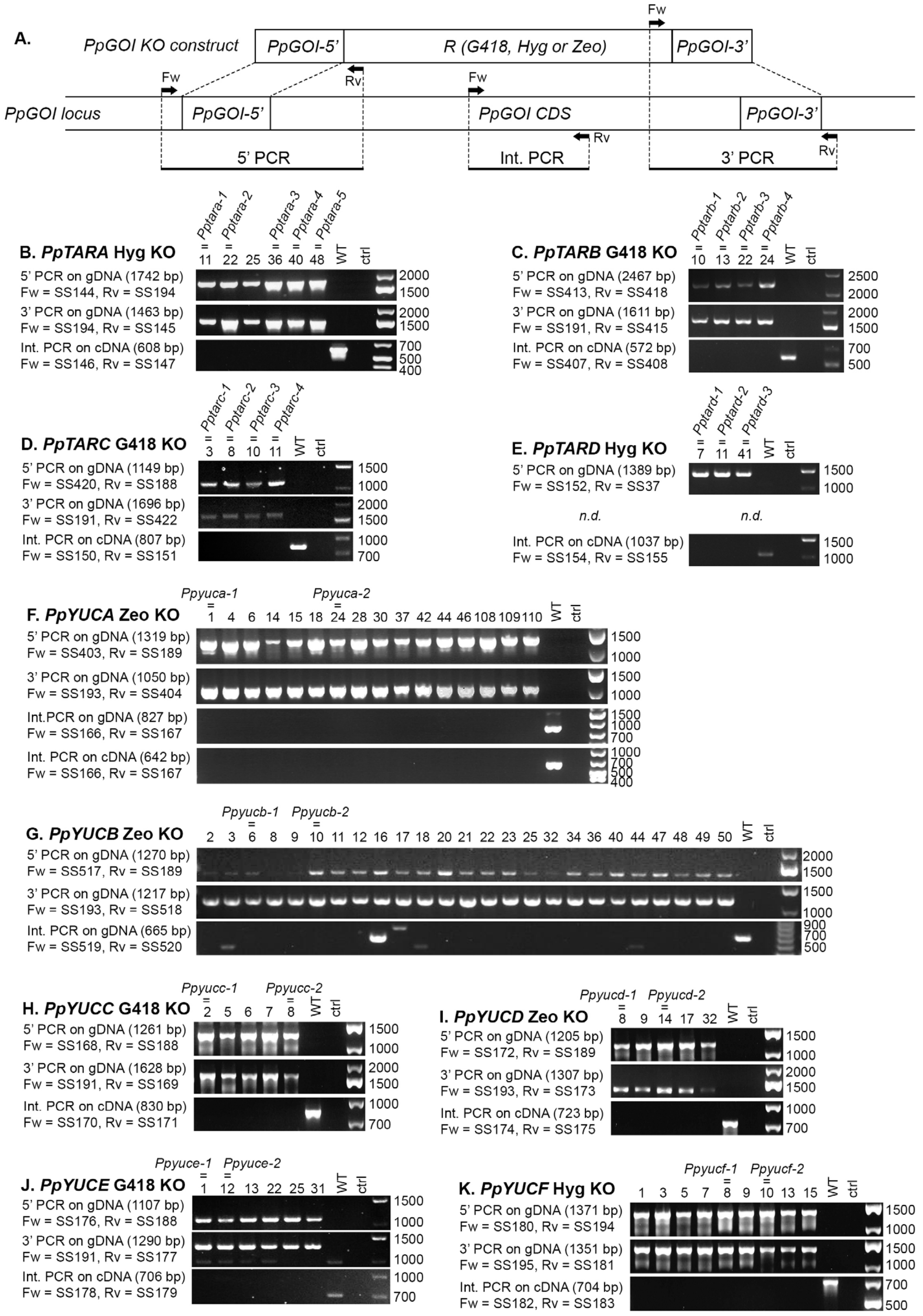

Fig. S5. PCR verification of *PpTAR* double and triple knockout lines. (**A**) PCR verification to confirm loss of *PpTARB* after transformation of the *Pptara-1* single mutant with the *PpTARB* KO construct pEP54. Primer names, expected product sizes and results are shown for PCRs confirming correct 5' junction, correct 3' junction, and the loss of internal gene sequences (see Fig. S4A for schematic explanation). (**B-D**) PCR verification to confirm the genotype of **(A)** *Pptaratarc* double mutants produced by a cross between *Pptara-1* and *Pptarc-4*, (**B**) *Pptarctard* double mutants produced by a cross between *Pptarc-4* and *Pptard-2*, and (**C**) *Pptarbtarctard* triple mutants produced by a cross between *Pptarb-2* and *Pptarctard-1*. In each case, primer names, expected product sizes and results are shown for a PCR confirming the loss of internal gene sequences (called Int. PCR in Fig. S4A). For primers sequences, see Table S2.

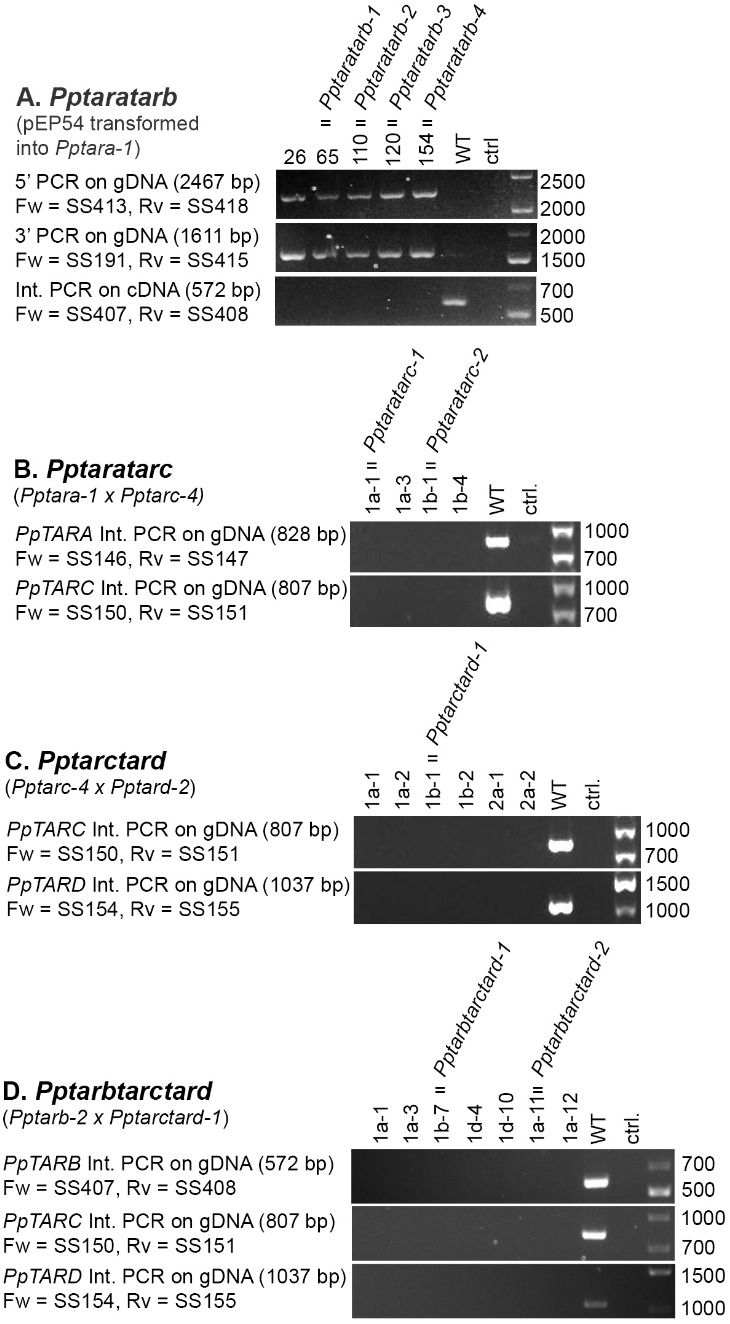

Fig. S6. Confirmation of knockout lines carrying the PpR2D2 reporter. Character matrix confirming that the selected meiotic offspring of crosses between the PpR2D2-2 and PpR2D2-3 auxin reporter lines (Thelander et al., 2019) and the *Pptara-1* and *Pptaratarc-1* lines, respectively, represent *Pptar* mutants carrying the PpR2D2 reporter. For primers sequences, see Table S2. Nd, not determined.

					Parent lines				
	PpR2D2-2 Pptara-1	*PpR2D2-3 Pptara-1*	*PpR2D2-2 Pptaratarc-1*	*PpR2D2-3 Pptaratarc-1*	*PpR2D2-2*	*PpR2D2-3*	*Pptara-1*	*Pptaratarc-1*	WT
Pptara phenotype	+	+	-	-	-	-	+	-	-
Pptara Pptarc phenotype	-	-	+	+	-	-	+	-	-
PpR2D2 signals	+	+	+	+	+	+	-	-	-
Hyg R (*PpTARA* KO construct)	+	+	+	+	-	-	+	+	-
G418 R (*PpTARC* KO and R2D2 constructs)	+	+	+	+	+	+	-	+	-
PpTARC locus deleted (no PCR product with internal *PpTARC* primers SS150 and SS151 on gDNA)	nd	nd	+	+	nd	nd	nd	+	-

Fig. S7. Intron positions in coding regions are conserved in Arabidopsis and *P. patens TAR* genes of both the TAA clade and the AtTAR3/4 clade. The alignment was produced from amino acid sequences deduced from primary models of all Arabidopsis (black) and *P. patens* (green) *TAR* genes with Clustal Omega using the default settings. Positions of introns spliced out to generate the coding sequences from which amino acid sequences were deduced are marked in red.

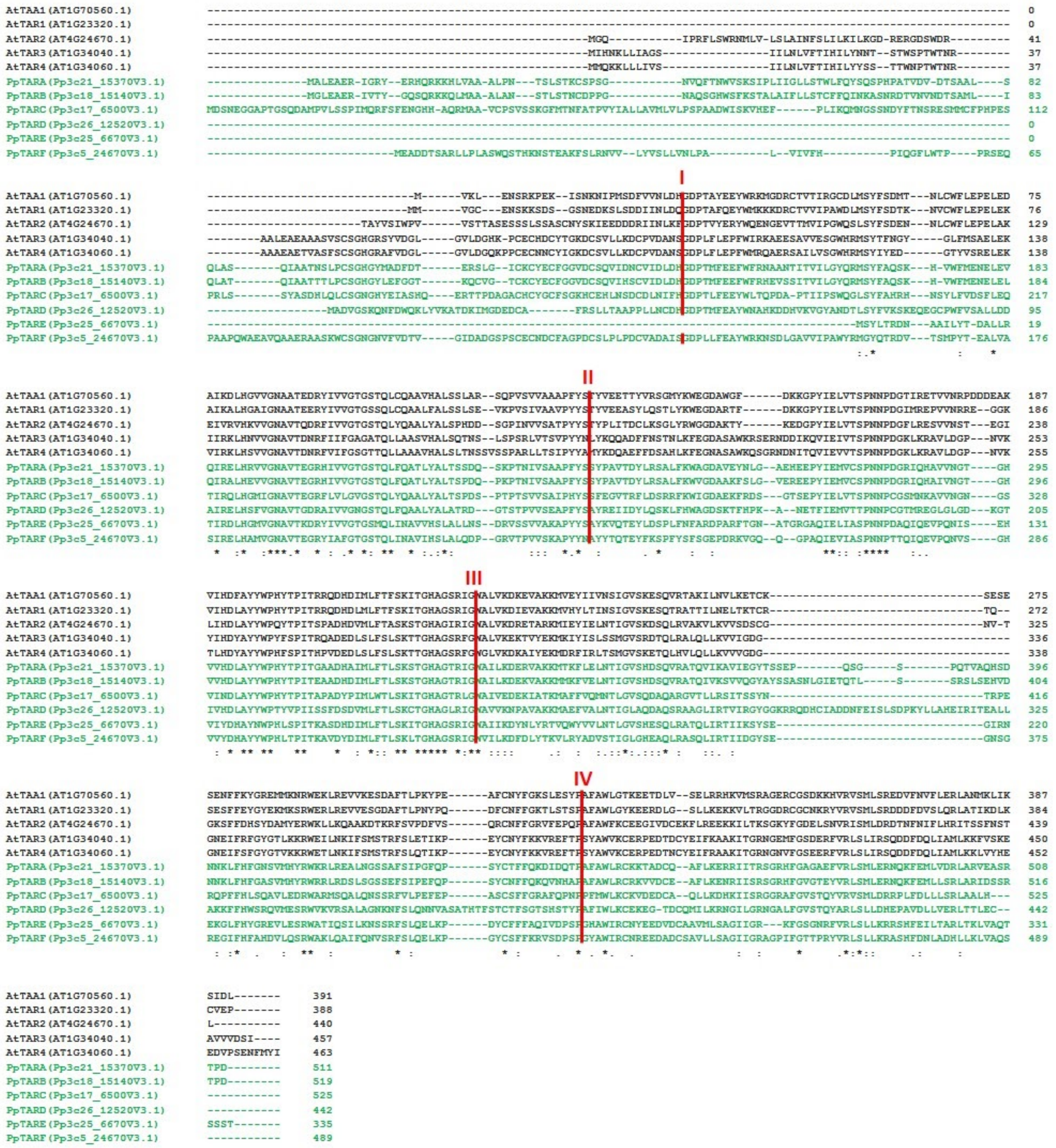

Fig. S8. The number of introns in *YUC* gene coding regions differ in both Arabidopsis and *P. patens* but the positions of introns that do exist are conserved. The alignment was produced from amino acid sequences deduced from primary models of all Arabidopsis (black) and *P. patens* (green) *YUC* genes with Clustal Omega using the default settings. Positions of introns spliced out to generate the coding sequences from which amino acid sequences were deduced are marked in red.

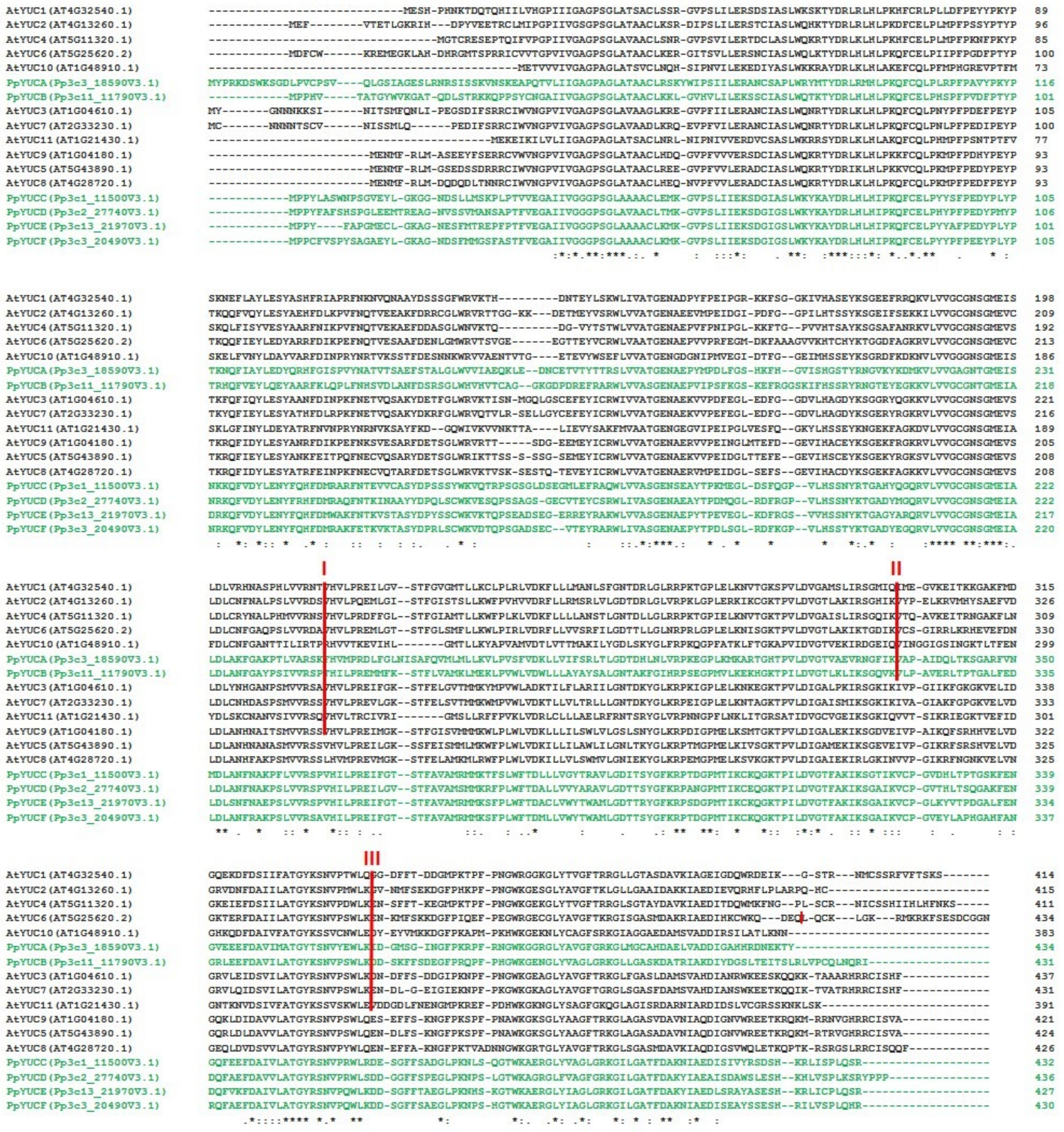

Fig. S9. The diameter of *Pptara* protonemal colonies is reduced. 21 day old colonies of three independent *Pptara* KO mutants and WT. Colony diameter is stated below each colony. Asterisks indicate statistically significant differences compared to WT, ** = $p < 0.01$ using Students t-test.

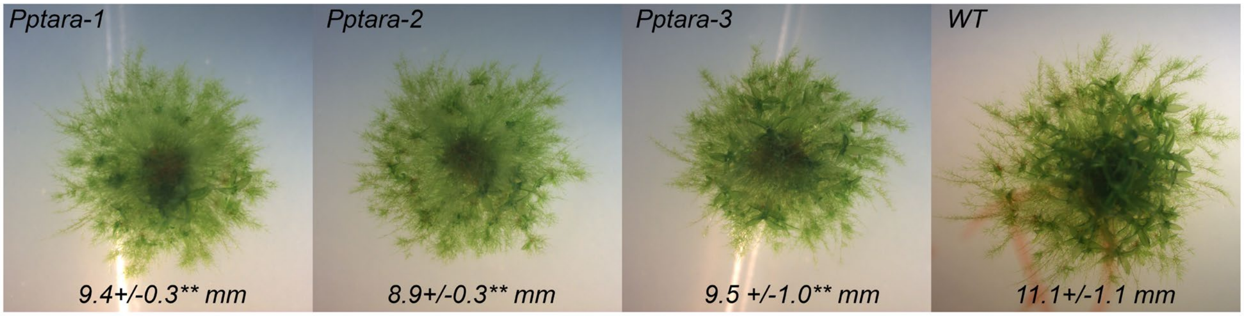

Fig. S10. Relative expression of *PpTAR* genes in chloronema. (**A**) Relative *PpTARA-D* transcript abundance in SPIA-amplified cDNA from chloronema-enriched protonema from the Reute ecotype. (**B**) Relative *PpTARA-D* transcript abundance in chloronema from the Gransden ecotype (eFP data from Ortiz-Ramirez et al., 2015). Error bars in (A) represent standard deviation of the mean of 3 biological replicates.

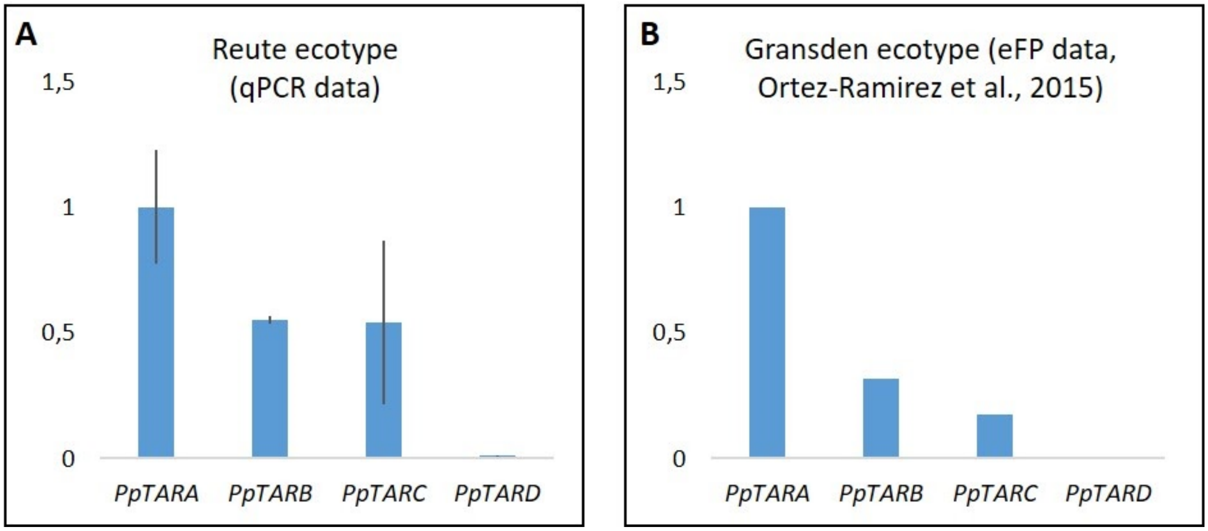

Fig. S11. *Pptaratarc* shoots are dwarfed but otherwise develop normally. Image shows representative adult shoots from WT, *Pptara-1*, *Pptarc-4*, *Pptaratarc-1, Pptaratarc-2* and WT grown for 30 days in standard growth conditions.

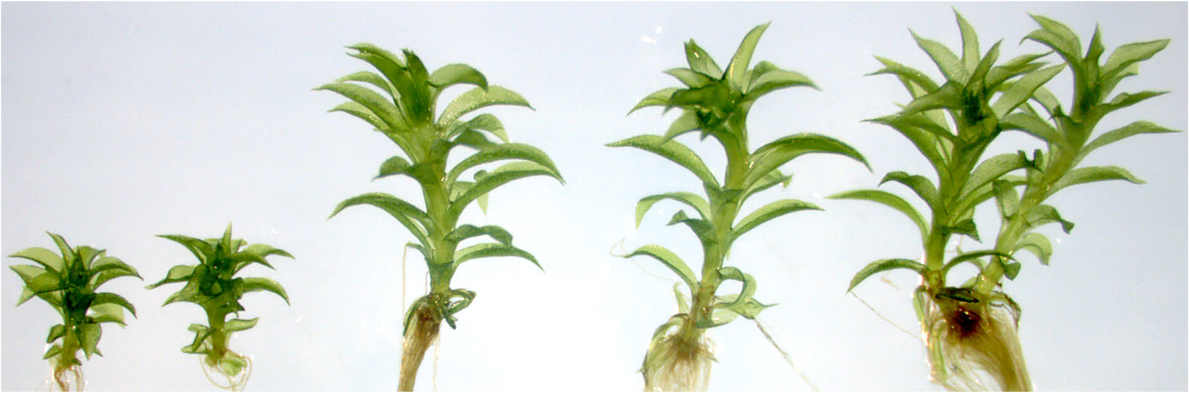

Pptaratarc-1 *Pptara-1* *Pptarc-4* *WT*
Pptaratarc-2

Fig. S12. Quantitative PpR2D2 output as a measure of auxin sensing during stage 2 antheridia development. Bar graphs showing average mDII-nVENUS:DII-nTdTOMATO signal ratios during stage 2 antheridia development as a measure of auxin sensing in (**A**) WT background (PpR2D2-3), (**B**) *Pptara* background (*PpR2D2-3 Pptara-1*) and (**C**) *Pptaratarc* background (*PpR2D2-3 Pptaratarc-1*). Errors bars indicate standard error of the mean. Asterisks indicate statistically significant difference between WT and mutant backgrounds (black) or between the two mutant backgrounds (red) in Student's t-tests ($P < 0.05$).

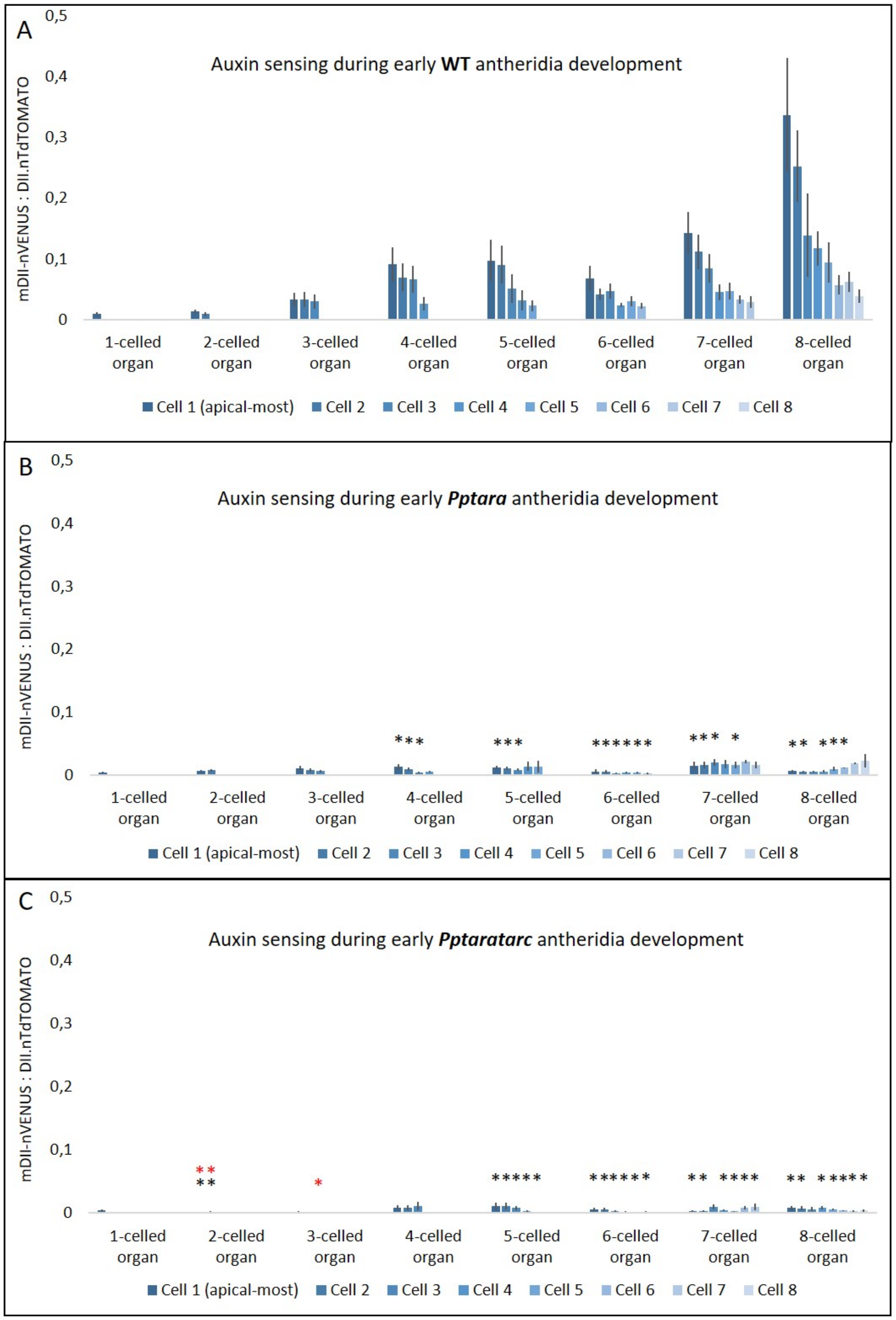

Fig. S13. Complementation of *Pptara* reproductive organ phenotype by *PpTARA* OE. The images show DIC micrographs of reproductive organs at comparable stages from the *Pptara-1* mutant and the *Pptara-1* mutant retransformed with the *ACT1pro::PpTARA* overexpressor construct. (**A**) *PpTARA* OE blocks the outgrowth of ectopic antheridia from the base of *Pptara* organs. Compare to Fig. 3e. (**B**) *PpTARA* OE blocks the formation of ectopic tip cells in *Pptara* antheridia. Compare to Fig. 4e. (**C**) *PpTARA* OE restores the formation of ectopic extra cells in the tip of *Pptara* archegonia. Compare to Fig. 6d.

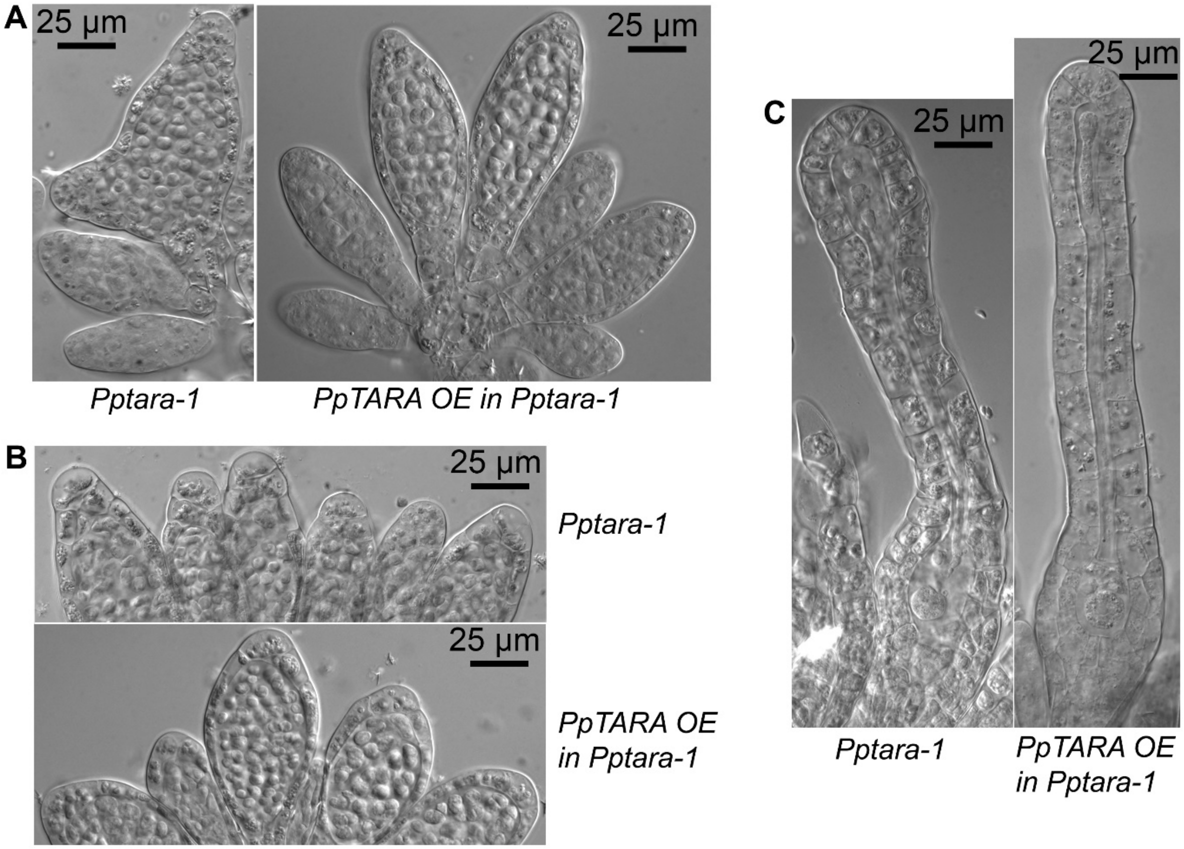

Fig. S14. Quantitative PpR2D2 output as a measure of auxin sensing during stage 3 to 9 antheridia development. Bar graphs comparing average mDII-nVENUS:DII-nTdTOMATO signal ratios in WT background (PpR2D2-3), *Pptara* background (*PpR2D2-3 Pptara-1*) and *Pptaratarc* (*PpR2D2-3 Pptaratarc-1*) background during stage 3 to 9 antheridia development as a measure of auxin sensing. (**A**) Cells in organ tip (all cells above inner cells considered). (**B**) Jacket cells (8 representative cells from layer surrounding inner cell cavity considered for each organ). (**C**) Inner cells (6 representative cells along apical-basal axis considered from each organ). Errors bars indicate standard error of the mean. Asterisks indicate statistically significant difference between WT and mutant backgrounds (black) or between the two mutant backgrounds (red) in Student's t-tests ($p < 0.05$).

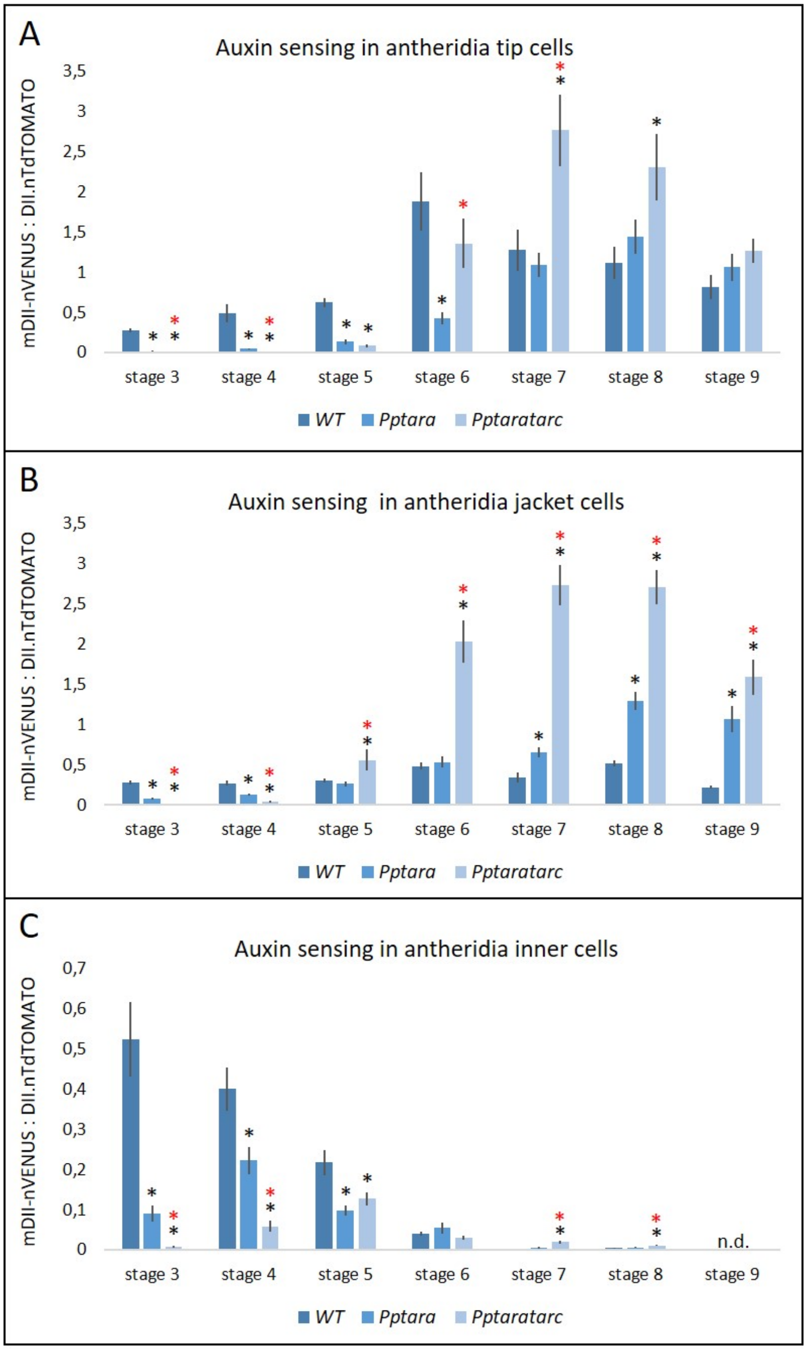

Fig. S15. A sub-set of inner cells do not proliferate in the *Pptaratarc* double mutant. The table summarizes the number of divisions that the six inner cells found in a stage 4 antheridium (see schematic organ drawing) have carried out when entering stage 6 in WT (right) and the *Pptaratarc-2* mutant (left). Each row represents an independent organ analyzed and each column represents a stage 4 inner cell of that organ (green numbers in column heading corresponds to green numbers in organ drawing and hence mark cell positions). Numbers shaded in pink report the number of divisions carried out by each stage 4 cell at the entrance of stage 6 development.

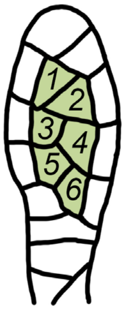

Pptaratarc-2

1	2	3	4	5	6
0	1	0	>2	2	0
0	>2	>2	>2	>2	
0	0	1	>2	>2	0
0	>2	>2	>2	>2	
0	>2	0	>2	0	>2
0	0	>2	>2	>2	
0	0	>2	>2	>2	>2
0	0	>2	>2	0	>2
0	0	0	>2	>2	>2
1	>2	>2	>2	>2	
1	1	>2	>2	0	0
0	>2	>2	>2	>2	0
>2	>2	>2	>2	>2	>2
0	1	0	1	>2	>2
>2	>2	0	0	>2	0
0	0	>2	0	>2	
0	1	>2	>2	>2	
0	0	0	>2	1	
1	2	>2	>2	>2	1
1	1	2	>2	1	0
0	>2	>2	>2	>2	
0	>2	>2	>2	0	0
1	>2	>2	>2	>2	
0	0	>2	>2	>2	>2
1	1	>2	>2		
0	0	0	>2	>2	0
0	0	>2	>2	>2	
0	0	0	>2	0	0
0	>2	0	>2	>2	
0	0	>2	0	0	0

WT

1	2	3	4	5	6
>2	>2	>2	>2	>2	>2
>2	>2	>2	>2	>2	>2
2	>2	>2	>2	>2	
>2	>2	>2	>2	>2	
>2	>2	>2	>2	>2	>2
>2	>2	>2	>2	>2	>2
1	>2	>2	>2	>2	
>2	>2	>2	>2	>2	
>2	>2	>2	>2		
>2	>2	>2	>2	>2	
>2	>2	>2	>2	>2	>2
>2	>2	>2	>2	>2	>2
2	>2	>2	>2	>2	>2
>2	>2	>2	>2	>2	>2
>2	>2	>2	>2	>2	>2
>2	>2	>2	>2	>2	>2
>2	>2	>2	>2	>2	
>2	>2	>2	>2	>2	>2
>2	>2	>2	>2	>2	>2
>2	>2	>2	>2	>2	>2
>2	>2	>2	>2	>2	
>2	>2	>2	>2	>2	>2
>2	>2	>2	>2	>2	>2
>2	>2	>2	>2	>2	>2
>2	>2	>2	>2	>2	>2
>2	>2	>2	>2	>2	>2
>2	>2	>2	>2	>2	>2
>2	>2	>2	>2	>2	>2
>2	>2	>2	>2	>2	>2
>2	>2	>2	>2	>2	>2

Fig. S16. Quantitative PpR2D2 output as a measure of auxin sensing during stage 1 and 2 archegonia development. Bar graphs showing average mDII-nVENUS:DII-nTdTOMATO signal ratios during stage 2 antheridia development as a measure of auxin sensing in (**A**) WT background (PpR2D2-2) and (**B**) *Pptara* background (*PpR2D2-2 Pptara-1*). Errors bars indicate standard error of the mean. Asterisks indicate statistically significant difference between WT and the mutant background in Student's t-tests (*, $p < 0.05$; **, $p < 0.02$).

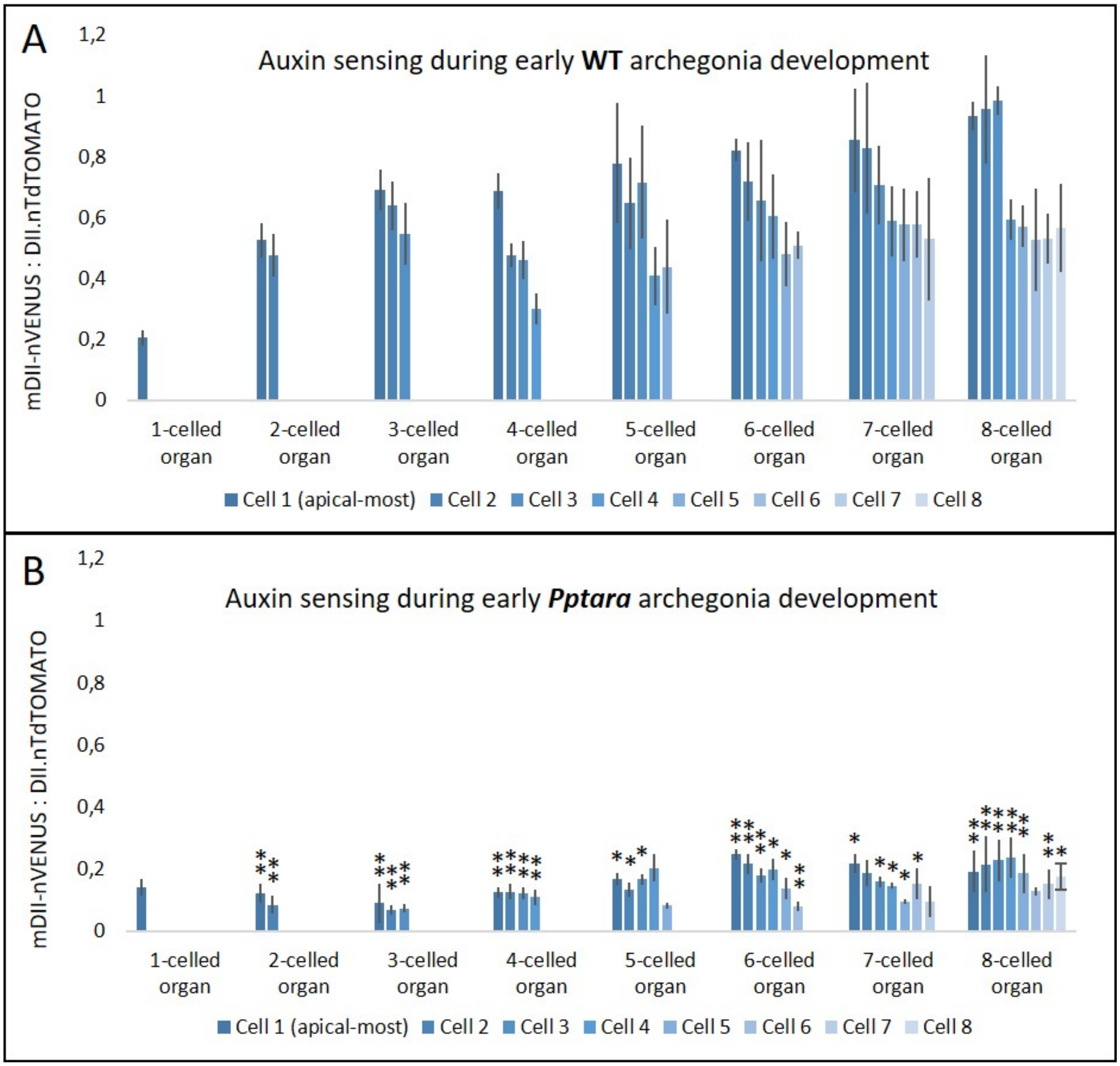

Fig. S17. Quantitative PpR2D2 output as a measure of auxin sensing during stage 3 to 9 archegonia development. Bar graphs comparing average mDII-nVENUS:DII-nTdTOMATO signal ratios in WT background (PpR2D2-2) and *Pptara* background (*PpR2D2-2 Pptara-1*) background during stage 3 to 9 archegonia development as a measure of auxin sensing. (**A**) Cells in organ tip (8 apical-most cells of each organ considered). (**B**) Inner cells (see schematic drawing in Fig. 6a). (**C**) Neck cells (6 cells in basal part of neck considered for each organ). Errors bars indicate standard error of the mean. Asterisks indicate statistically significant difference between WT and the *Pptara* mutant backgrounds in Student's t-tests ($p < 0.05$).

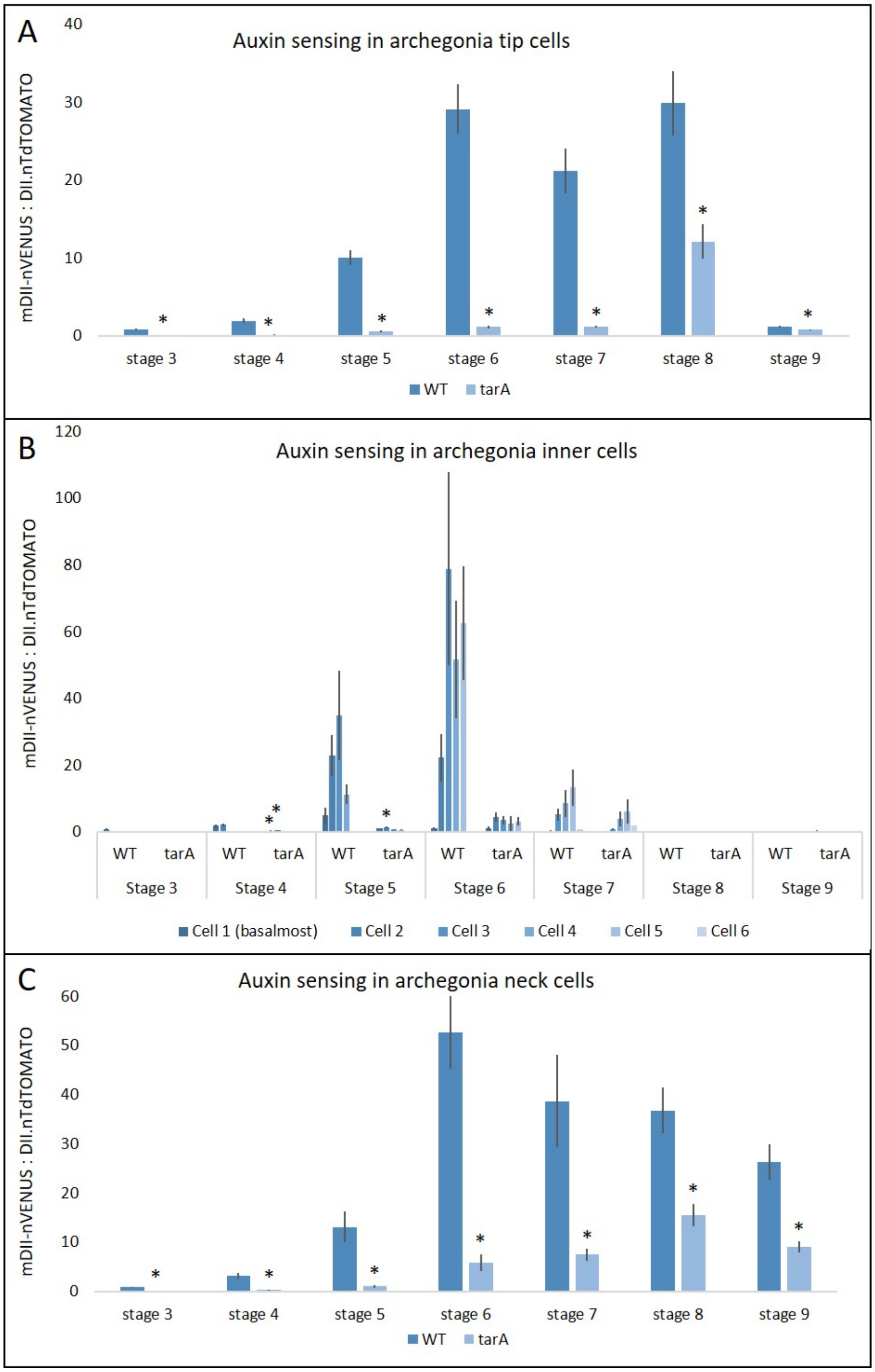

Fig. S18. Archegonia neck lengths are reduced in *Pptar* mutants due to a cell elongation defect. (**A**) Bar graph showing average neck lengths in archegonia in different developmental stages from *Pptara-1*, *Pptaratarb-1*, *Pptaratarc-2* and WT. (**B**) Bar graph showing average number of cells in the outermost row of cells in the neck of archegonia in different developmental stages from *Pptara-1*, *Pptaratarb-1*, *Pptaratarc-2* and WT. Error bars mark standard deviation and asterisks mark a statistically significant difference to WT in Student's t-tests (* = $p < 0.05$; ** = $p < 0.01$).

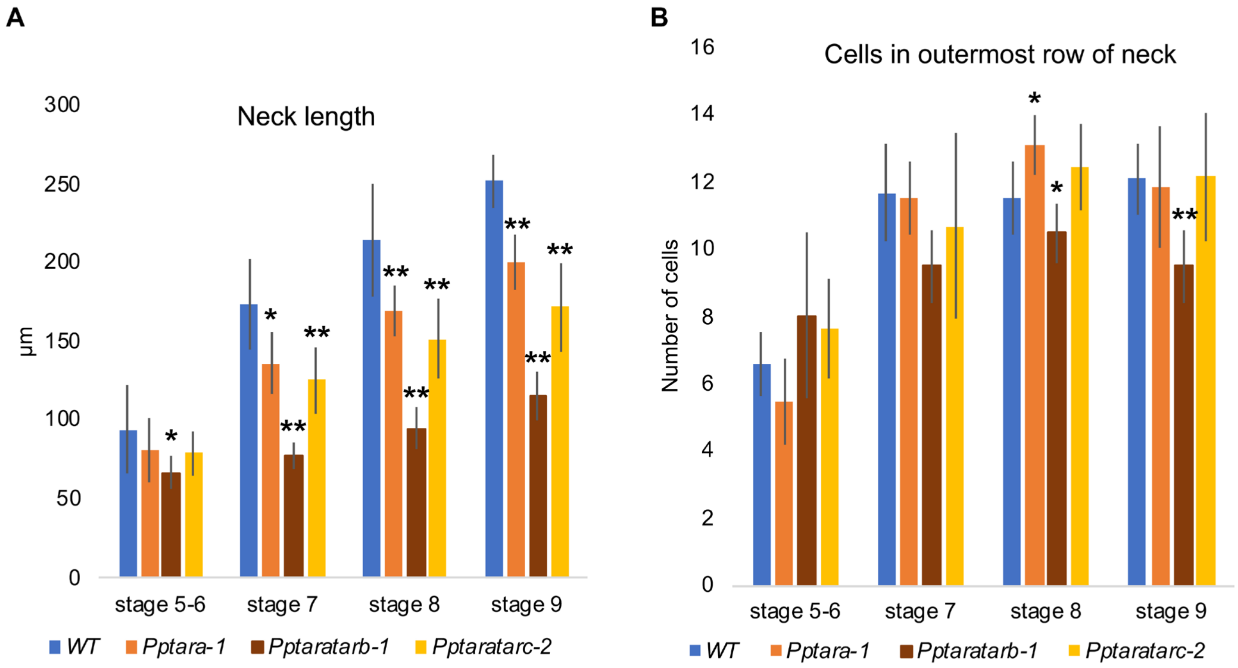

Fig. S19. ***PpYUC* expression patterns and knockout phenotype resemble those of *PpTAR* in reproductive organs. (A)** Representative micrographs of the *Ppyucb* mutant showing a phenotype resembling that of *Pptar* mutants. The leftmost image shows a mature reproductive apex where antheridia and archegonia have been false colored in yellow and red, respectively. Compare to WT in Fig. 3c and note the hyperformation of antheridia and the stunted archegonia with fleshy tips. The middle image shows a close-up of a late stage antheridium with borders of tip cells marked for clarity. Compare to WT in Fig. 4e and note ectopic tip cells. The rightmost image shows a close-up of a stage 8 archegonium with inner cells and their borders shaded in yellow for clarity. Compare to stage 8 WT in Fig. 6d and note the stunted appearance of the organ, the extra outer cells in the organ tip and the wide and irregularly shaped apical cavity. (**B)** Representative examples of *PpYUCF::GFPGUS* reporter output in antheridia of various stages. For each stage, a merge of confocal channels detecting GFP (green) and chloroplast autofluorescence (red) is shown to the left and DIC image to the right. Compare to *PpTAR::GFPGUS* reporter output in Fig. 2f VII and Fig. 4b and note the largely overlapping expression pattern. (**C)** Representative examples of *PpYUCF::GFPGUS* reporter output in mid-stage archegonia. For each stage, a merge of confocal channels detecting GFP (green) and chloroplast autofluorescence (red) is shown to the left and DIC image to the right. Compare to *PpTARA::GFPGUS* reporter output in Fig. 6b and note the largely overlapping expression pattern.

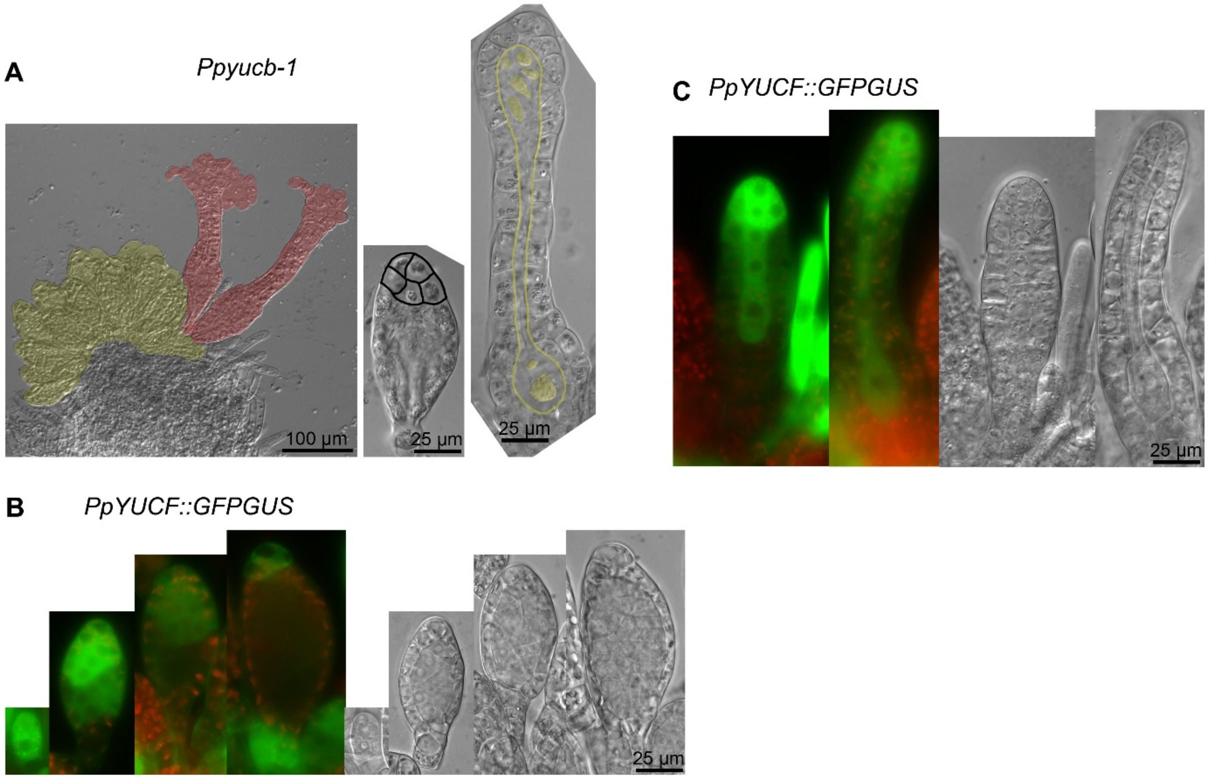

Fig. S20. **Auxin sensing in the apical stem cell of young vegetative leaves is successively increased in a *PpTAR*-dependent manner.** Bar graphs showing PpR2D2 output (average mDII-nVENUS:DII-nTdTOMATO signal ratios) as a measure of auxin sensing in cells along a single proximo-distal cell file of early vegetative leaves with different numbers of cells along the proximo-distal axis. (**A**) WT background (PpR2D2-3). (**B**) *Pptara* background (*PpR2D2-3 Pptara-1*). (**C**) *Pptaratarc* background (*PpR2D2-3 Pptaratarc-1*). The leftmost bar (darkest blue) in each cluster represents the apical cell. Errors bars indicate standard error of the mean. Asterisks indicate statistically significant difference between WT and the *Pptaratarc* mutant backgrounds in Student's t-tests ($p < 0.05$). The fact that both *PpTARA* and *PpTARC* must be deleted for reduced auxin sensing is in line with the observation that both genes are expressed in young leaves (Fig. 2d,f; Fig. 5b).

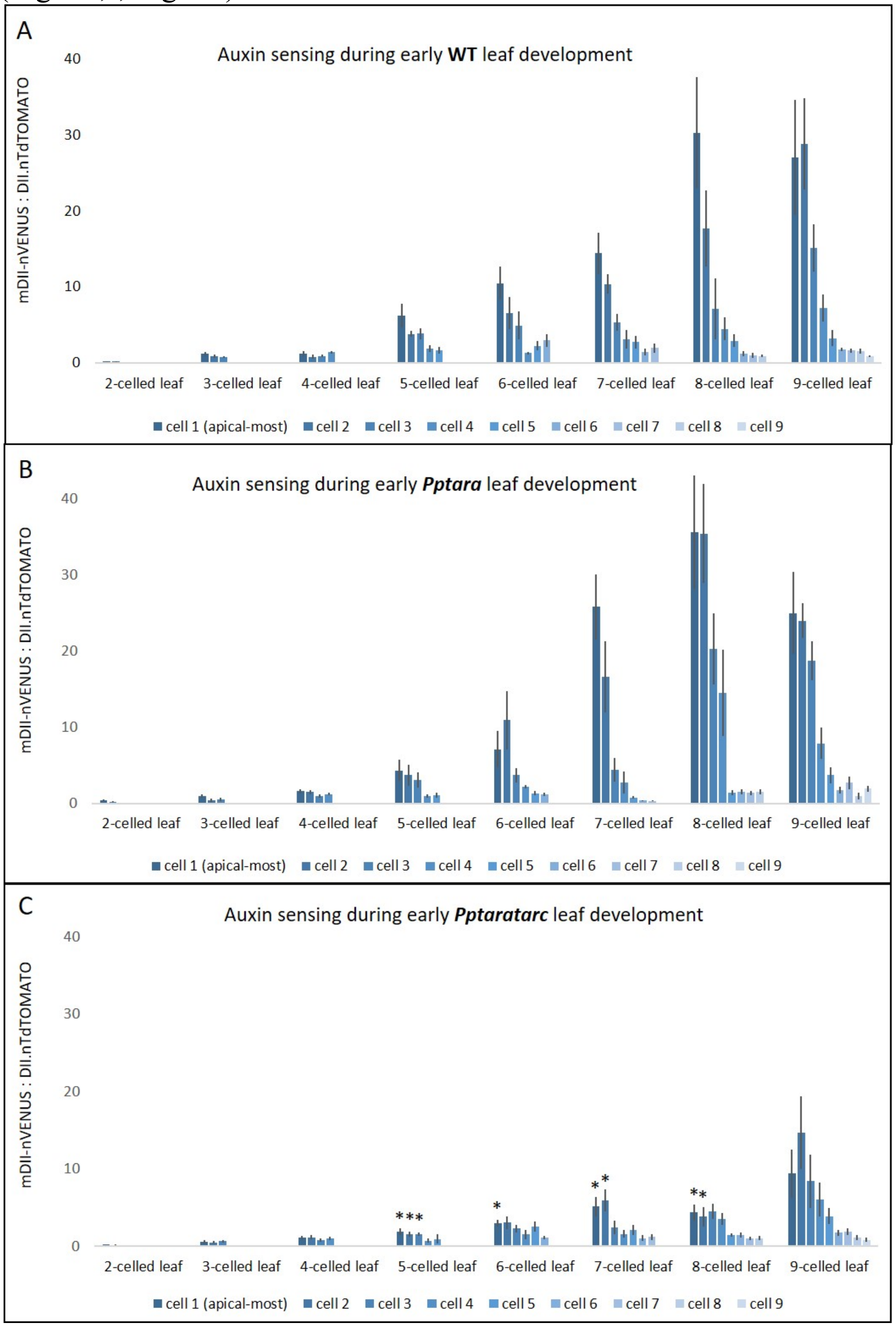

Fig. S21. ***PpPIND* is expressed in spermatogenous cells of antheridia.** Representative epifluorescence micrographs of signal output from the *PpPINDpro::GFP-1* transcriptional reporter (Viaene et al., 2014). **(A)** Antheridia bundle with organs in different developmental stages. **(B)** Mid-stage antheridium at high magnification. In both (A) and (B), a merge of channels detecting GFP (green) and chloroplast autofluorescence (magenta) as well as a DIC image are shown.

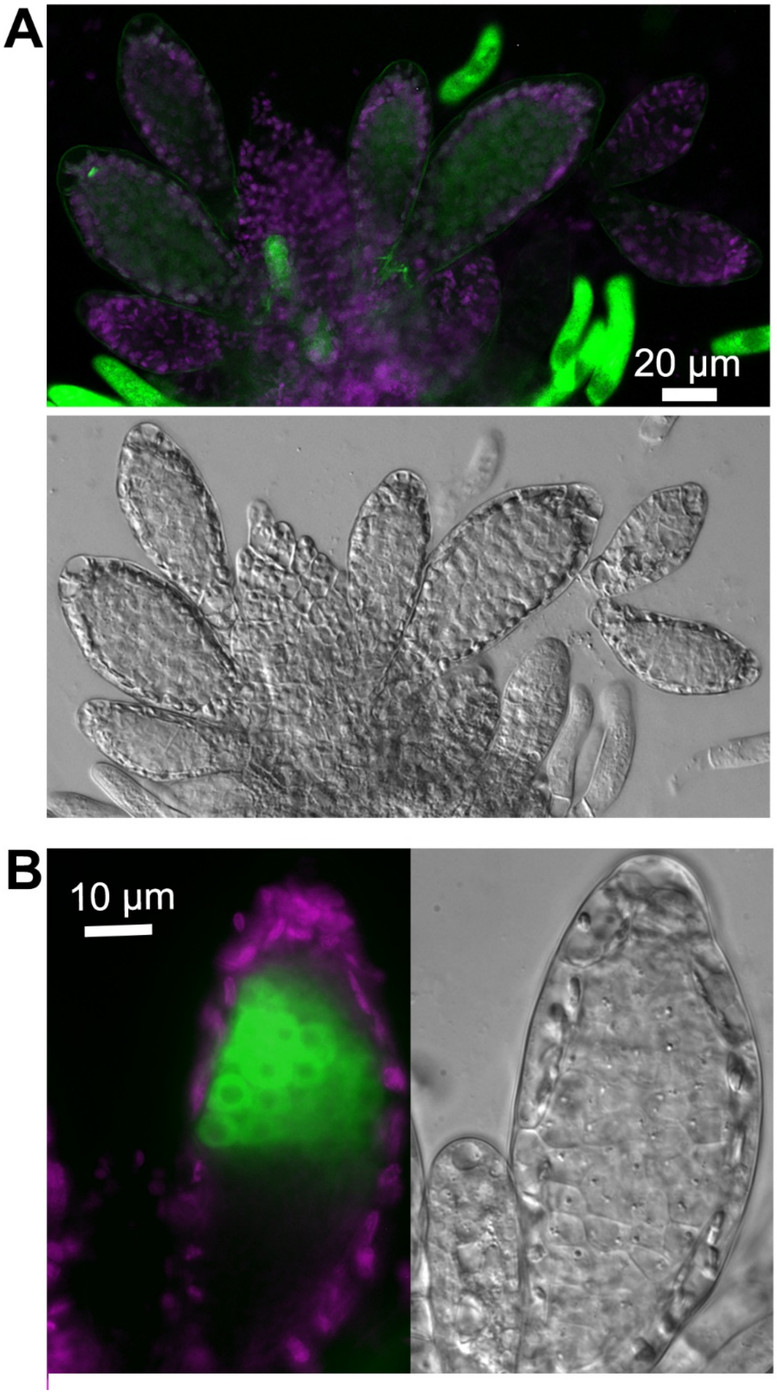

Fig. S22. **Long *PpPINs* are expressed in the pre-egg / egg from around stage 5 of archegonia development.** Representative micrographs of signal output from long *PpPIN* reporters in the body of archegonia at selected developmental stages. S3/5/6/7/8/9 indicate developmental stages (Fig. 6a; Landberg et al., 2013). **(A)** *PpPINB::GFPGUS-1* reporter output from stage 4 to stage 9. **(B)** *PpPINC::GFPGUS-1* reporter output from stage 6 to stage 9. **(C)** *PpPINA::PpPINA-GFP-2* reporter output from stage 6 to stage 8. In (A) and (B), a merge of confocal channels detecting GFP (green) and chloroplast autofluorescence (magenta) is shown on top and a DIC image is shown below for each stage. In (C), to better visualize the weak and dotty GFP signal produced by the translational reporter, the confocal channel detecting chloroplast autofluorescence is omitted. Instead, a long exposure of the confocal channel detecting GFP (green) is shown on top and a DIC image is shown below.

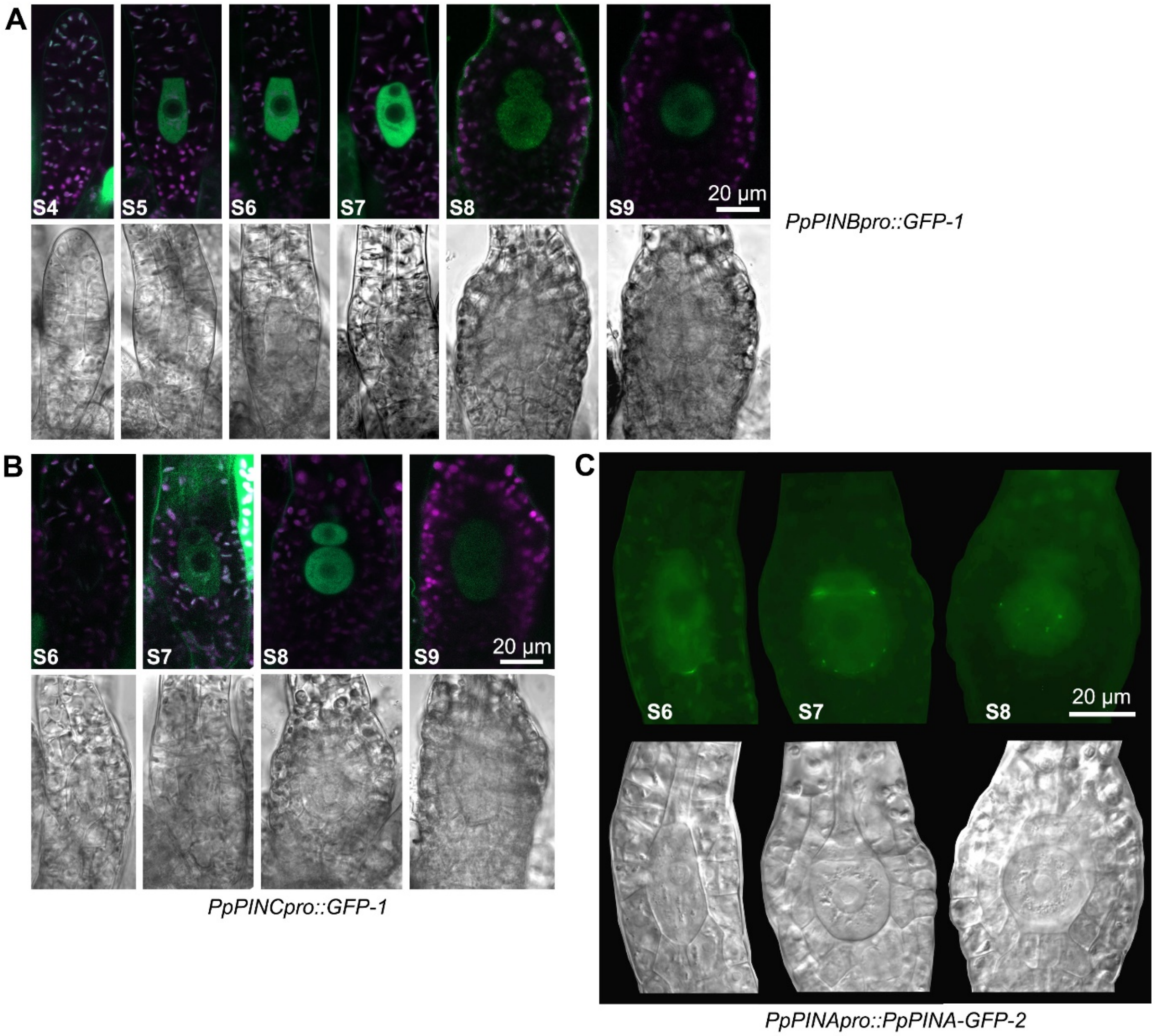

Table S1. Constructs produced and used in the study

**Name**	**Description**
pDONR4r-3r-G418	G418R entry clone
pDONR4r-3r-Zeo	ZeoR entry clone
pEP54	*PpTARB* KO construct (G418R)
pMT207	*PpTARD* KO construct (HygR)
pMT210	*PpTARA* KO construct (HygR)
pMT222	HygR entry clone
pMT229	*PpTARC* KO construct (G418R)
pMT232	*PpYUCC* KO construct (G418R)
pMT233	*PpYUCE* KO construct (G418R)
pMT235	*PpYUCF* KO construct (HygR)
pMT236	*PpYUCD* KO construct (ZeoR)
pMT237	*PpTARD* trx reporter (HygR)
pMT241	*PpTARB* trx reporter (HygR)
pMT243	*PpTARA* trx reporter (HygR)
pMT246	*PpYUCF* trx reporter (HygR)
pMT254	*PpTARC* trx reporter (HygR)
pMT258	OE vector (pACT1, G418R)
pMT261	*PpTARF* OE construct (G418R)
pMT262	*PpYUCC* OE construct (G418R)
pMT263	*PpYUCA* KO construct (ZeoR)
pMT266	*PpYUCA* OE construct (G418R)
pMT272	OE vector (pACT1, ZeoR)
pMT276	*PpYUCB* KO construct (ZeoR)
pMT281	*PpTARA* OE construct (ZeoR)

Table S2. Primers used in this study

PCR primers used to build constructs	
**Name**	**Sequence**
SS220	CGCGGATCCATCAGGAGTTG
SS221	GAGCCATGGCGGAGTCGATAAGGCGACTCAG
SS222	TTGTCATGAGCTGCATCGAAGCCGCAC
SS223	GACTCATGATGGCATCGACGAAGTGAGAAAATG
SS224	AAGGGATCCGAGAGGGTGGTGATGGTGGTGG
SS225	TGTCCATGGTTGCAGACAGACAGTAGCTTGTGCG
SS226	TATAAGCTTGAGGGATCTTGGTTTGAATCTTGGT
SS227	CTGCCATGGTGATCAGTACCCGGGATGGTCC
SS244	CATGGATCCTCTTAATGTACAGCTGCTTTCATCTCC
SS245	TGACCATGGTGATCGATTGAGGTGTTCACCGA
SS265	GGGG ACA ACT TTT CTA TAC AAA GTT G ATGGCGCAGGGGATCAAGATCA
SS266	GGGG AC AAC TTT ATT ATA CAA AGT TGT CCCCCGAGGGGATCGA
SS269	GGGGACAAGTTTGTACAAAAAAGCAGGCTCTCGAGGTCGCCCCGCGTGGAGAGGT
SS270	GGGGACAACTTTGTATAGAAAAGTTGGGTGAATTTTATTCAAGAGGGCGTTCCTGA
SS271	GGGGACAACTTTGTATAATAAAGTTGCATCCAATGTACACAGATTTCAGTTCC
SS272	GGGGACCACTTTGTACAAGAAAGCTGGGTACTCGAGTAATTTTGAGGTTTTGGAGTAACACCA
SS281	GGGGACAAGTTTGTACAAAAAAGCAGGCTCTCGAGCATGAAGGGGATTTGAAAGCATAA
SS282	GGGGACAACTTTGTATAGAAAAGTTGGGTGGAATCGCTGGTCGCTGTGATGAAA
SS283	GGGGACAACTTTGTATAATAAAGTTGTGCCAGCCTTTAAAATCCCTCAAC
SS284	GGGGACCACTTTGTACAAGAAAGCTGGGTACTCGAGCTGGCAAGCGCGGTAACG
SS285	GGGGACAACTTTGTATAGAAAAGTTGGGTGCTGGCGAGTGGGAGAATGCAAAGTAG
SS286	GGGGACAAGTTTGTACAAAAAAGCAGGCTCTCGAGTCTAGACGCAATGAATGAGCGAAC
SS287	GGGGACAACTTTGTATAATAAAGTTGCTGAATCCCCAGTCCGTCACTA
SS288	GGGGACCACTTTGTACAAGAAAGCTGGGTACTCGAGTTCGATGTTCCAATGTTGTTGTTTAC
SS290	GGGGACAAGTTTGTACAAAAAAGCAGGCTGTCGACTTAGCCCTCACCGTTGTCAAGTCCTC
SS292	GGGGACAACTTTGTATAGAAAAGTTGGGTGAAGAGTAGTTCTAACCACCGAGATGG
SS293	GGGGACAACTTTGTATAATAAAGTTGCGCCCTCTGTGCAGCACCATTCC
SS294	GGGGACCACTTTGTACAAGAAAGCTGGGTAGTCGACCGAGCCATCGCTGTGTACCAAGACTG
SS295	GGGGACAACTTTGTATAGAAAAGTTGGGTGATCGCTGCAATGGGTCGTCTGAACC
SS296	GGGGACAAGTTTGTACAAAAAAGCAGGCTGTCGACCTCATTCAGGCCCGTGGTTTGGTG
SS298	GGGGACAACTTTGTATAATAAAGTTGTCGGCATTTGCGGAAGGAGTTTGAG
SS300	GGGGACCACTTTGTACAAGAAAGCTGGGTAGTCGACGGGCGGACGGTAGTGATGGTGGTT
SS325	GGGGACAAGTTTGTACAAAAAAGCAGGCTGTCGACGATGCCGAACCAGCCACAAG
SS326	GGGGACAACTTTGTATAGAAAAGTTGGGTGTGGTCAATTGTGCACCTACAAGC

SS368	GGGGACAACTTTGTATAATAAAGTTGGTATGCGGTCGGTTTCGGTAGGAA
SS369	GGGGACCACTTTGTACAAGAAAGCTGGGTAGTCGACTTGGCGTTTTTGGATAGAGCAGTGATT
SS370	CAACCTAGGATGTACCCCAGAAAGGATTCTTGG
SS371	TTTGTCGACTCAATAGGTCTTTTCATTATCTCTGTGG
SS372	ACTCCTAGGATGCCGCCCTACCTTGCTTC
SS373	CAGCCTAGGCTATCTAGACTGGAGCGGGGAGATG
SS378	TCGCCTAGGATGGAGGCCGATGACACGTC
SS379	GAGGTCGACTCAAGATTGAGCGACGAGTTTGAG
SS424	GGGGACAAGTTTGTACAAAAAAGCAGGCTGTCGACAATGGCCCCCACAAAAGCAGTTA
SS425	GGGGACAACTTTGTATAGAAAAGTTGGGTGTGAGCAGAGGTTCTACCCCAGCATA
SS426	GGGGACAACTTTGTATAATAAAGTTGCCCGAGGGAGAACCCATCGTT
SS469	ATCCCTAGGATGGCTCTTGAAGCCGAGCG
SS470	CATCCTAGGCTAGTCGGGGGTTCTGGAAGCC
SS476	GGGGACCACTTTGTACAAGAAAGCTGGGTAGTCGACGTGAGAGGCTCTCTCTGCAGCG
SS596	AAGAAGCTTCGAGGTCATTCATATGCTTGAGAAG
SS598	CTAACTAGTGTCGACCCTAGGTCTACCTACAAAAAAGCTCCGCAC
SS599	ATCCTCGAGCTGTGAGACACAATGTTGTTCA
SS600	ATCTCAAGCTTCTTGATCAGTACCCGGGATGGT
SS601	ACAACTAGTGAAGATTAGCACGACTG
SS602	AAAGAGCTCAACAACAATAAGATATTA
SS603	CGCTATCTCGAGAAGTGGTGGACTTGATGAGTG
SS604	CGCTATAAAGCTTTCGGAGTCGATAAGGCGACT
SS605	CGCTATTCTAGACCCATGCCTCGCTTGTTGG
SS606	CGCTATTGAGCTCAATGTTGCTGCTGAGGCC
SS607	TCGCTATGGATCCTTTTGGCAGGTTGGGGACG
SS608	CGCTATCTCGAGATTGGCATCGACGAAGTGAGAAA
SS609	CGCTATACTAGTGCGTAGTTCTTCATGCCTCAAGTC
SS610	CGCTATATTAATTAAGCCAGGACGGTGATCATAGT
SS611	GGGGACAACTTTTCTATACAAAGTTGAATTCCCATGGAGTCAAAG
SS612	GGGGACAACTTTATTATACAAAGTTGTAATTCGAGCTCGGTACCCAC
SS613	GGGGACAACTTTTCTATACAAAGTTGAATTCCCTTTCAGAAAGAATG
SS614	GGGGACAACTTTATTATACAAAGTTGTCCGTCACCGGTGTGAGGGAAC
**Genotyping primers**	
**Name**	**Sequence**
SS5	GAAACCTCCCAAGCTCTGACGA
SS37	CGGCTGAGTGGCTCCTTCAA
SS144	CGGCTGGCTGCGTTCCTAT
SS145	GAGGGCAATCATTTTCTGGAGTGG

SS146	TTCCGAAACGCAGCTAACACTATCAC
SS147	AACTTCGTCATCTTCTTCGCCACTCT
SS150	GTACGCCTTAACATCACCCGACTCC
SS151	GCTCATCCTGGCCCACCTATCT
SS152	GGTTTTCGCTCCATACTCTCGTCTA
SS154	GATCGTGGTCGGTAATGGAAGC
SS155	CCGAAAAAGTGCAAGTGGAAAAC
SS166	CGCGCCGCTTTGGAGATA
SS167	CAAGCGTAAGCCTGGAGAAGATGAC
SS158	TGAGCCTTCAACTTTAATCGAATAGACT
SS169	AGTGACCTAGTATGTGGCGGGGATTA
SS170	CGGCGTGCCTGGAGATGAA
SS171	GCCCGGGCACACCTTGATG
SS172	ACCCAGCCCACAACCTCACAT
SS173	GGATGACATTCTTGCACTTTTTACACA
SS174	CTACTTTCGTCGAGGGTGCCATTAT
SS175	CCAGAAGCGCGTCCGTGAA
SS176	GGCCCGCTCATCCTCTTTTG
SS177	AGTTTCCCCACATGGTCGTCACA
SS178	GAGGGAGTACCGGGCGAAGTG
SS179	CAACCCGGCGATGTAGAGACC
SS180	GCGGAGGTCAGGGCTTAGG
SS181	GATCCATCAAGCACCACATTCCTA
SS182	GGGGTTTCGACAGGGTGGTTTA
SS183	ACAGGCGCGGGTCGTATGA
SS188	AGTCTTTACGGCGAGTTCTGTTAGGT
SS189	CATCTGTGGGTTAGCATTCTTTCTG
SS191	AGGCATGCCCGCTGAAATC
SS193	TCAAGATCAAAACTAGTTCCCTCACACC
SS194	ACGCGCAATAATGGTTTCTGAC
SS195	ACACCGAGCGGCGAACTAATAA
SS307	CGTCCGAGGGCAAAGAAATAGAGTA
SS308	GCTCGGCACAAAATCACCACTC
SS348	AGGCTGTGGTATGGGCTTGTCTAAC
SS354	GCGGCGCTTTATCCTTCCACTAC
SS355	GCTCGGTTCTCCTGGGGAAGTAGT
SS356	AGGCAGCGCTGAGGTTTCATTTA
SS357	TGCAGATTGTCCCTTTTCCTTCACTA

SS398	GGAGGGATCAAACGTAACAGAACAA
SS400	ACCTGCGCTTTCAAGCCTCACT
SS401	TTCACTTTGGGCCACCTTTTATTACC
SS402	CTGACCGCTTCCTCGTGCTTTAC
SS403	CGGGCAATCTTCCTCTTCTTCTAC
SS404	ACTATGCTTGGGGATCTTGAAATGGA
SS407	ATATTGTCTCTGCCGCTCCGTTCTA
SS408	TCGCCACTTTCTCGTCTTTCAGG
SS413	GACTTGACCATGTGGAGGGACTTC
SS415	ATTTGGCAGGCGTGGAAGG
SS418	GAGGAAGGGTCTTGCGAAGGATAGTG
SS420	CTTCTTCATGCCCTCTTTCGGATAC
SS422	CACTTTAGTCGTAGGTTTCCGTCAGGTA
SS517b	GGAAACCCAACAGGAGCACTTACTA
SS518b	CCCCTCCTTCACTCCTGCTCTG
SS519	CCCACCCGCCACCAGTTC
SS551	CGTGGCCGAGGAGCAGGAC
**qPCR primers**	
**Name**	**Sequence**
SS29	CGGAGAGGAAGTACAGTGTGTG
SS30	ACCAGCCGTTAGAATTGAGCC
SS428	AGTTTCGTGCGCAGTGGTTGG
SS429	ACTCGCTGCCCCTGGTAATGG
SS430	CCGACTCTCGTAGCTCGCAGTAA
SS431	CGAGACCGGCAACACTTTCAAAA
SS434	GGGCATGCTGGGAGTCGTATT
SS435	CCCTGCCTGAGTTGCCTTCC
SS455	TACGGACCCTAATCCAGATGAC
SS456	CAACCCATTGCATACTTCTGAG
SS459	AGTATAGTCTAGAGTATGGTACCG
SS460	TAGCAATTTGATGGCAGCTC
SS463	GTCTAGTTAGTCCTTTGGTCCT
SS464	GCCTATTTCTATAATGACTCCGT
SS501	ACTCGAATTGGGTGGGCTATCTTGA
SS502	CATTTGTGGCTCGGACGAGGTGTAT
SS503	CACGCATTGGGTGGGCTATCC
SS504	CCCTGGACTACACTCTTCACAATCTGC

SS517	ACTCGCCTTGGTTGGGCTATTG
SS518	TCTCTGGCCGCGTGTTGTAACTA
SS519	ATGCCATCAGGGAGCTTCACTCAT

Table S3. Level of auxin metabolites in wildtype *P. patens* and in stated over-expressor (OE) and mutant lines, expressed as mmol per gram fresh weight. The mean values are based on 5-6 independent biological replicates, except for wildtype where 20 samples were analysed. UDL = under detection limit. Asterisks indicate statistically significant differences compared to WT, * = p < 0.05 and ** = p < 0.01 using Students t-test. Error values represent standard deviation. The WT data presented here is the same data as in Table 1 in actual manuscript.

Abbreviation	Compound	WT	*PpTARA OE-1*	*PpTARA OE-2*	*PpTARF OE-1*	*PpTARF OE-2*	*PpYUCA OE-1*
TRP	Tryptophan	21785 ± 5712	10855 ± 2421**	11659 ± 2193**	22830 ± 4694	19387 ± 2922	14700 ± 2930*
TRA	Tryptamine	11,77 ± 3,56	10,67 ± 2,74	9,25 ± 1,95	8,81 ± 4,92	7,13 ± 3,83*	10,22 ± 5,42
IAN	Indole-3-acetonitrile	UDL	UDL	UDL	UDL	UDL	UDL
IAM	Indol-3-acetamide	UDL	UDL	UDL	UDL	UDL	UDL
IPyA	Indole-3-pyruvic acid	82,99 ± 17,22	764,60 ± 152,30**	1583,90 ± 336,53**	99,21 ± 16,22	83,48 ± 20,79	48,74 ± 6,37**
IAA	Indol-3-acetic acid	8,50 ± 1,75	40,90 ± 7,75**	41,70 ± 2,53**	8,40 ± 2,26	6,65 ± 1,59*	13,25 ± 2,18**
oxIAA	2-oxoindole-3-acetic acid	27,17 ± 5,16	55,45 ± 5,39**	59,78 ± 14,82**	23,29 ± 7,83	31,24 ± 7,28	46,17 ± 5,52**
OxIAA-Glc	oxIAA-glucose	46,69 ± 12,41	42,65 ± 11,85	72,15 ± 17,99**	32,86 ± 14,66*	41,98 ± 18,92	29,06 ± 12,39*
IAA-Glc	IAA-glucose	7,84 ± 2,08	2,57 ± 1,08**	4,19 ± 1,78**	4,13 ± 0,82**	7,69 ± 1,17	8,21 ± 1,71
IAA-Asp	IAA-aspartate	0,28 ± 0,09	UDL	0,45 ± 0,25	0,21 ± 0,04	0,26 ± 0,11	0,16 ± 0,04*
IAA-Gly	IAA-glycine	0,58 ± 0,24	0,41 ± 0,15	0,47 ± 0,09	1,09 ± 0,29**	1,01 ± 0,11**	1,51 ± 0,28**
IAA-Glu	IAA-glutamate	0,62 ± 0,15	0,39 ± 0,10*	0,47 ± 0,17	0,17 ± 0,09**	0,67 ± 0,05	1,29 ± 0,21**
IAA-Val	IAA-valine	UDL	UDL	UDL	UDL	UDL	UDL
IAA-Leu	IAA-leucine	0,08 ± 0,03	0,13 ± 0,02**	0,14 ± 0,03**	0,05 ± 0,01*	0,05 ± 0,02	UDL
IAA-Phe	IAA-phenylalanine	UDL	UDL	UDL	UDL	UDL	UDL
IAA-Trp	IAA-tryptophan	UDL	UDL	UDL	UDL	UDL	UDL

Abbreviation	Compound	*PpYUCA OE-2*	*PpYUCC OE-1*	*PpYUCC OE-2*	*Pptara-1*	*Pptarb-1*	*Pptaratarb-1*
TRP	Tryptophan	12205 ± 2507**	15188 ± 1680*	12482 ± 1383**	26461 ± 10518	16699 ± 2564	33330 ± 5050**
TRA	Tryptamine	8,24 ± 4,44	7,73 ± 4,07*	8,89 ± 5,27	14,15 ± 5,17	7,42 ± 1,55**	12,60 ± 5,73
IAN	Indole-3-acetonitrile	UDL	UDL	UDL	UDL	UDL	UDL
IAM	Indol-3-acetamide	UDL	UDL	UDL	UDL	UDL	UDL
IPyA	Indole-3-pyruvic acid	29,45 ± 10,44**	45,91 ± 12,15**	28,70 ± 11,22**	15,88 ± 2,79**	62,88 ± 12,32*	13,34 ± 1,19**
IAA	Indol-3-acetic acid	9,86 ± 3,00	15,99 ± 3,67**	10,52 ± 1,13*	4,13 ± 1,63**	6,04 ± 0,85**	2,71 ± 0,65**
oxIAA	2-oxoindole-3-acetic acid	60,95 ± 7,71**	68,86 ± 6,93**	70,38 ± 4,23**	15,70 ± 2,44**	31,78 ± 10,69	6,54 ± 1,92**
OxIAA-Glc	oxIAA-glucose	29,07 ± 10,66**	28,97 ± 11,74**	34,76 ± 9,34	42,44 ± 17,62	28,40 ± 8,47*	46,62 ± 14,19
IAA-Glc	IAA-glucose	6,94 ± 2,07	8,65 ± 2,18	9,27 ± 1,82	5,78 ± 2,05	4,85 ± 1,56**	6,11 ± 0,78
IAA-Asp	IAA-aspartate	0,15 ± 0,07**	0,21 ± 0,07	0,24 ± 0,10	UDL	UDL	UDL
IAA-Gly	IAA-glycine	1,45 ± 0,19**	1,51 ± 0,17**	1,02 ± 0,06**	0,48 ± 0,11	0,35 ± 0,10*	0,45 ± 0,09
IAA-Glu	IAA-glutamate	0,99 ± 0,15**	1,43 ± 0,18**	1,25 ± 0,27**	UDL	UDL	UDL
IAA-Val	IAA-valine	UDL	UDL	UDL	UDL	UDL	UDL
IAA-Leu	IAA-leucine	0,03 ± 0,00**	0,05 ± 0,02*	UDL	0,10 ± 0,04	0,07 ± 0,02	0,11 ± 0,03
IAA-Phe	IAA-phenylalanine	UDL	UDL	UDL	UDL	UDL	UDL
IAA-Trp	IAA-tryptophan	UDL	UDL	UDL	UDL	UDL	UDL

Supplementary references

Landberg K, Pederson ERA, Viaene T, Bozorg B, Friml J, Jönsson H, Thelander M, Sundberg E. 2013. The moss *Physcomitrella patens* reproductive organ development is highly organized, affected by the two *SHI*/*STY* genes and by the level of active auxin in the *SHI*/*STY* expression domain. *Plant Physiology* **162**: 1406-1419.

Le Bail A, Scholz S, Kost B. 2013. Evaluation of reference genes for RT qPCR analyses of structure-specific and hormone regulated gene expression in Physcomitrella patens gametophytes. *PLoS One.* **8:** e70998.

Ortiz-Ramirez C, Hernandez-Coronado M, Thamm A, Catarino B, Wang M, Dolan L, Feijo JA, Becker JD. 2016. A transcriptome atlas of *Physcomitrella patens* provides insights into the evolution and development of land plants. *Molecular Plant* **9**: 205-220.

Prigge MJ, Lavy M, Ashton NW, Estelle M. 2010. *Physcomitrella patens* auxin-resistant mutants affect conserved elements of an auxin-signaling pathway. *Current Biology* **20:** 1907–1912.

Thelander M, Olsson T, Ronne H. 2004. Snf1-related Protein Kinase 1 Is Needed for Growth in a Normal Day-Night Light Cycle. *EMBO J.* **23:** 1900-1910.

Thelander M, Nilsson A, Olsson T, Johansson M, Girod PA, Schaefer DG, Zryd JP, Ronne H. 2007. The moss genes *PpSKI1* and *PpSKI2* encode nuclear SnRK1 interacting proteins with homologues in vascular plants. *Plant Molecular Biology* **64:** 559–573.

Thelander M, Landberg K, Sundberg E. 2019. Minimal auxin sensing levels in vegetative moss stem cells revealed by a ratiometric reporter. *New Phytologist* **224**: 775-788.

Viaene T, Landberg K, Thelander M, Medvecka E, Pederson E, Feraru E, Cooper ED, Karimi M, Delwiche CF, Ljung K, Geisler M, Sundberg E, Friml J. 2014. Directional auxin transport mechanisms in early diverging land plants. *Current Biology* **24**: 2786-2791.
